# Optimised techniques for high-throughput screening of differentiated SH-SY5Y cells and application for neurite outgrowth assays

**DOI:** 10.1038/s41598-021-03442-1

**Published:** 2021-12-14

**Authors:** Anusha Dravid, Brad Raos, Darren Svirskis, Simon J. O’Carroll

**Affiliations:** 1 School of Pharmacy, Faculty of Medical and Health Sciences, University of Auckland, Private Bag 92019, Auckland, New Zealand; 2 Department of Anatomy and Medical Imaging, School of Medical Sciences, Faculty of Medical and Health Sciences, University of Auckland, Private Bag 92019, Auckland, New Zealand

**Keywords:** Biological techniques, Neuroscience

## Abstract

Neuronal models are a crucial tool in neuroscientific research, helping to elucidate the molecular and cellular processes involved in disorders of the nervous system. Adapting these models to a high-throughput format enables simultaneous screening of multiple agents within a single assay. SH-SY5Y cells have been widely used as a neuronal model, yet commonly in an undifferentiated state that is not representative of mature neurons. Differentiation of the SH-SY5Y cells is a necessary step to obtain cells that express mature neuronal markers. Despite this understanding, the absence of a standardised protocol has limited the use of differentiated SH-SY5Y cells in high-throughput assay formats. Here, we describe techniques to differentiate and re-plate SH-SY5Y cells within a 96-well plate for high-throughput screening. SH-SY5Y cells seeded at an initial density of 2,500 cells/well in a 96-well plate provide sufficient space for neurites to extend, without impacting cell viability. Room temperature pre-incubation for 1 h improved the plating homogeneity within the well and the ability to analyse neurites. We then demonstrated the efficacy of our techniques by optimising it further for neurite outgrowth analysis. The presented methods achieve homogenously distributed differentiated SH-SY5Y cells, useful for researchers using these cells in high-throughput screening assays.

## Introduction

Neurons are responsible for transmitting electrical action potentials that elicit sensory and motor responses. Many disorders of the central nervous system (CNS) have been attributed to the degeneration, trauma, or dysfunction of these cells^1^. In vitro neuronal cell culture models are crucial tools in neuroscientific research, enabling a deeper understanding into the cellular and molecular basis that underlies physiological and pathophysiological processes of the CNS in a more controlled, reproducible environment^1–3^. Neurite outgrowth is an example of a key physiological process of interest, due to its fundamental role in both neurodevelopment and regeneration after traumatic injury^4^. Hence, neurite outgrowth assays are widely employed to screen the growth promoting or inhibitory effects of chemical compounds on neuronal cells^5^. By developing these assays in a high-throughput screening format, researchers can test a large number of soluble factors in an efficient and automated manner^6^.

A number of in vitro models have been described in the literature including: cells derived from primary tissue^7^, immortalised cell lines^8^, human induced pluripotent stem cells (iPSCs)^9^, and explant/organotypic cultures. Of particular interest to this paper, immortalised cell lines have potential for large-scale expandability, and can be effectively adapted for high-throughput screening assays. Despite the caveat of a tumorigenic origin, the use of these cells is attractive due to their human source, ease of handling compared to iPSCs, and potential for rapid expandability.

This paper focuses on the human SH-SY5Y cell line, which is a subclone of the parental neuroblastoma SK-N-SH cell line. SH-SY5Y cells have been widely employed as an in vitro neuronal model for neurodegeneration^10^, neurotrauma^11^, developmental neurotoxicity^12^ and neurite outgrowth^13^. However, they have most commonly been used in an undifferentiated state^14^. Undifferentiated cells have a tendency to grow in clusters with only several, short projections. Moreover, undifferentiated cells do not express mature neuronal markers. Hence, differentiation is a necessary step to produce a population of cells with morphological and functional behaviours likened to that of mature neurons. Such properties include the ability to fire action potentials, express neuronal receptors, and exhibit a dependency on neurotrophic factors^15,16^. There are a number of different protocols reported in the literature to differentiate SH-SY5Y cells into a neuronal phenotype^13,15–18^. Depending on the reagents used, these cells can be differentiated to possess dopaminergic, cholinergic or adrenergic properties^3^. As such, SH-SY5Y cells have broad applicability in the study of neurobiology.

Despite their popularity, the use of differentiated SH-SY5Y cells for high-throughput screening has been limited. One reason for this is the absence of a standardised culture protocol^3^. The overall goal of this paper was to address this need for a defined protocol for differentiating SH-SY5Y cells in a high-throughput format. We detail an approach to effectively differentiate the SH-SY5Y cell line into a neuronal phenotype within a 96-well plate. Additionally, we have optimised the seeding density for downstream neurite outgrowth analysis. We then report the effects of different detachment reagents and the successful re-plating of differentiated SH-SY5Y cells. Re-plating terminally differentiated cells enables study of neurite outgrowth, regeneration and synapse assembly in post-mitotic cells^19^. Finally, we demonstrate the efficacy of our protocol in a high-throughput neurite outgrowth assay of nerve growth factor (NGF), brain-derived neurotrophic factor (BDNF) and neurotrophin-3 (NT-3), as differentiated SH-SY5Y cells are known to possess receptors for these growth factors^20^.

## Methods

### SH-SY5Y maintenance culture and differentiation

SH-SY5Y cells (ATCC, CRL-2266) were differentiated and cultured using a combination of previously reported protocols^15,16^ (Supplementary Fig. S1). Our simplified approach ensures cells are exposed to the appropriate medium supplements required for terminal differentiation into a neuronal phenotype without multiple passaging steps. This simplicity is necessary for efficient differentiation within a high-throughput format. Briefly, undifferentiated cells were maintained in basic growth medium, comprised of Dulbecco’s Modified Eagle’s Medium (DMEM) (Gibco Life Technologies, #11965-092), supplemented with heat-inactivated foetal bovine serum (hiFBS) (Moregate, #3827104), 1 × glutamax-I (Gibco Life Technologies, #35050-51) and 1 × penicillin/streptomycin (Gibco Life Technologies, #15646-055). To induce differentiation, cells were seeded onto Matrigel-coated (1:100 dilution) (Corning, #FAL356231) dishes at the specified densities relevant to the experiment. The following day, the medium was changed to Stage I medium comprised of DMEM supplemented with hiFBS (2.5%), 1 × glutamax-I, 1 × penicillin/streptomycin and retinoic acid (RA) (10 μM) (Sigma, R2625, Sigma Aldrich, MO, USA). After five days, the medium was changed to Stage II medium comprised of Neurobasal-A media (Gibco Life Technologies, #10888-022) supplemented with brain-derived neurotrophic factor (BDNF) (50 ng/mL) (Novus Bio/R&D Systems, #248-BD), potassium chloride (KCl) (20 mM) (Sigma, #P5405), 1 × B27 (Gibco Life Technologies, #17564-044), 1 × glutamax-I and 1 × penicillin/streptomycin for an additional five days. Cultivated cells were maintained below passage 12 at 37 °C/5% CO_2_. Phase contrast images of the cells at different stages of differentiation were acquired daily using the EVOS FL microscope equipped with a 10 × or 20 × objective.

### Investigating different room temperature pre-incubation periods on cell plating homogeneity

Achieving an even distribution of plated cells within each well is an important parameter that can affect both cell behaviour, microscopic imaging and subsequent analyses. Room temperature pre-incubation is one approach to achieve uniformity, by enabling cells to attach before transfer into the incubator^21^. Undifferentiated SH-SY5Y cells were plated onto Matrigel-coated 96-well plates (n = 3 replicate wells) at a density of 10,000 cells/well by dispensing the cell suspension into the centre of the well. Plates were incubated for either 15 min, 30 min, or 1 h in a Class II biological safety cabinet at room temperature. Control plates were immediately transferred to the incubator after plating (i.e. no pre-incubation). After 24 h, cells were fixed using 4% paraformaldehyde (PFA) and labelled with a Hoechst nuclear stain (1:10,000 dilution). A full well scan of each well was acquired at 10 × magnification using the EVOS FL Auto microscope (Life Technologies). A custom FIJI^22^ script was used to analyse the percentage cell occupied area in five different segmented regions as indicated in Supplementary Fig. S2. The script has been provided in the supplementary information. The efficacy of pre-incubation was determined by comparing the standard deviation of the cell occupied area between each of the regions, with a lower standard deviation between the different regions corresponding to increased plating homogeneity.

### Antibodies

The following antibodies and dilutions were used: mouse-anti-βIII-tubulin (Sigma, #T8660, 1:400), mouse-anti-Gap-43 (Invitrogen, #33-5000, 1:50), mouse-anti-Tau (Sigma, #T9450, 1:100) and Anti-mouse-Alexa Flour 488 (Abcam, #ab150105, 1:500).

### Immunocytochemistry

SH-SY5Y cells were fixed by initially removing half the culture media and adding in an equal volume of 4% paraformaldehyde (PFA) (#441244, Sigma Aldrich) in PBS at pH 7.4, to achieve a concentration of 2% PFA in the well. All incubations were at room temperature, unless otherwise stated. After a 2 min incubation, this solution was aspirated, and the cells were incubated for an additional 10 min in 4% PFA solution. Cells were then washed with PBS for 1 min before permeabilization using PBS-Triton (0.1%) for 10 min. Next, cultures were blocked with 2% bovine serum albumin (BSA) (#ABGF-100G, MP Biomedicals) dissolved in PBS-Triton (0.1%) for 1 h, and washed with PBS-Tween 20 (0.1%) three times for five minutes. Cells were then incubated with the primary antibody diluted in 1% BSA/PBS-Triton (0.1%) overnight at 4 °C. After washing with PBS-Tween 20 (0.1%) followed by a 1 h incubation with the secondary antibody and Hoechst nuclear counterstain solution (1:10,000 dilution).

### Determining a suitable plating density in for neurite outgrowth in a 96-well plate format

Undifferentiated cells were plated in three different Matrigel-coated 96-well plates at densities of 1,250 cells/well, 2,500 cells/well, 5,000 cells/well, 10,000 cells/well, 15,000 cells/well. The first plate was fixed using 4% PFA at the start of differentiation (day 1), prior to the addition of stage I media, and labelled with Hoechst nuclear counterstain (n = 3 replicate wells for each density). The second plate was fixed at the end of differentiation (day 11), for labelling with mouse-anti-βIII-tubulin antibody and a Hoechst nuclear counterstain (n = 3 replicate wells for each density). Four images per well were acquired using an inverted EVOS FL Auto microscope equipped with a 20 × objective lens. βIII-tubulin images were acquired using the GFP filter (excitation 482/25 nm; emission 524/24 nm), while Hoechst nuclei images were acquired using the DAPI filter (excitation 357/44 nm; emission 447/60 nm). The nuclei were manually counted using the cell counter plugin on FIJI^22^ to quantify the increase in cell number over the differentiation period. The third plate was used to perform a LIVE/DEAD viability/cytotoxicity test at the end of differentiation (day 11) to assess the viability of the cells at different plating densities (n = 5 replicate wells).

### Comparison of different detachment reagents for re-plating differentiated SH-SY5Y cells

SH-SY5Y cells plated in a 12-well plate (50,000 cells/well) were differentiated according to our protocol described above. At day 11, cells were detached using either trypsin–EDTA (0.05%), Accutase (#7920, StemCell Technologies), Dispase (#07923, StemCell Technologies), Versene (#15040-055, Gibco life technologies) or gentle cell dissociation reagent (GCDR) (#07174, StemCell Technologies) as detailed in Supplementary Table S1 and re-plated in 96-well plates at a density of 10,000 cells/well for immunocytochemistry and viability analysis.

### LIVE/DEAD viability/cytotoxicity assay

Cells were incubated with the LIVE/DEAD Viability/Cytotoxicity reagents (Invitrogen, #L3224) according to the manufacturer’s protocol. Briefly, Calcein-AM and ethidium homodimer-1 (EthD-1) stock solutions were diluted in Neurobasal media to achieve final concentrations of 2 µM and 4 µM respectively. Cells were rinsed with 1 × PBS and incubated with the Calcein-AM/EthD-1 solution for 1 h at 37 °C/5% CO_2_. Images were acquired using the EVOS FL Auto microscope equipped with a 10 × or 20 × objective where specified, using the GFP (Calcein-AM) or Texas Red (EthD-1) filters. A fluorescence microplate reader (Varioskan LUX, Thermo Scientific) was used to obtain fluorescence emission measurements for Calcein-AM (ex: 485, em: 530) and EthD-1 (ex: 530, em: 645). Background fluorescence of Matrigel and the culture media was also measured at each wavelength and subtracted from the obtained readings.

### High-throughput neurite outgrowth assay

Our optimised differentiation protocol was then validated by screening the effects of NGF, BDNF and NT-3 in a high-throughput neurite outgrowth assay under four different conditions. These soluble factors were selected due to their established role in promoting neurite outgrowth within the central and peripheral nervous systems. Additionally, it has been reported that RA promotes upregulation of neurotrophin receptors^20,23^. The neurite outgrowth assay was performed using SH-SY5Y cells that were (i) terminally differentiated, (ii) terminally differentiated and in the presence of soluble chondroitin sulfate proteoglycan (CSPG) neurite outgrowth inhibitors, and (iii) re-plated using trypsin–EDTA (0.05%) after terminal differentiation. This has been visually illustrated in Supplementary Fig. S3, where the bands indicate the time points in the differentiation protocol in which the dose–response assay was started for each of the different cell stages. For all dose–response assays, NGF (#NBP2-36437, Novus Bio), BDNF (#248-BD, R&D Systems) and NT-3 (#450-03, Peprotech) were added to Stage II medium to achieve a final concentration of 12.5 ng/mL, 25 ng/mL, 50 ng/mL, 100 ng/mL, 200 ng/mL or 400 ng/mL, whilst control wells did not contain any neurotrophins. SH-SY5Y cells were plated at an initial density of 2,500 cells/well in 96-well plates. For terminally differentiated cells, the neurotrophins were added after five days in Stage II medium (day 11—end of differentiation). To evaluate the effects of the neurotrophins on neurite outgrowth in the presence of growth inhibitory molecules, the dose–response assay for terminally differentiated cells was repeated in the presence of pure extracellular CSPGs (#CC117, Merck Millipore) at a concentration of 10 µg/mL in each well. This concentration of soluble CSPG was selected from the study by Jin et al^24^. For terminally differentiated cells after re-plating, cells were differentiated and re-plated using trypsin–EDTA (0.05%) and a dose–response assay performed as described above. Plates were incubated for either 24 h or 48 h in the neurotrophic medium. At each of these designated time points, cells were fixed in 4% PFA and labelled with mouse-anti-βIII-tubulin antibody and a Hoechst nuclear counterstain (1:10,000 dilution). Images (16-bit) were acquired in TIF format using an inverted EVOS FL Auto microscope with a 20 × objective lens. βIII-tubulin images were acquired using the GFP filter (excitation 482/25 nm; emission 524/24 nm), while Hoechst nuclei images were acquired using the DAPI filter (excitation 357/44 nm; emission 447/60 nm). Each concentration was investigated in triplicate wells, with three images acquired per well. Prior to analysis of the neurite networks, the nuclei and corresponding neurite images were assigned to separate colour channels and merged to form a composite image using FIJI software. Analysis of the neurite networks was then performed on the composite images using the MorphoNeuroNet FIJI macro, designed by Pani et al.^25^. In our study, we quantified neurite outgrowth as the ratio of total neurite length to the number of nuclei to account for any differences in cell number between images.

### Statistical analyses

Statistical analyses were performed using GraphPad Prism 8.0.2 for Windows (GraphPad Software, San Diego). All results have been presented as the mean ± standard deviation (SD). A one-way ANOVA test was used to evaluate statistical significance, as indicated by a *p*-value < 0.05. The Shapiro–Wilk test indicated residuals showed normality. Post hoc testing was conducted using Tukey multiple comparisons.

## Results and discussion

### Differentiated SH-SY5Y cells express neuronal markers and morphological features of mature neurons

In the present paper, we combined two differentiation protocols^15,16^ to obtain terminally differentiated neurons within a 96-well plate. The rationale behind combining these two protocols was to obtain a more efficient approach to differentiate the SH-SY5Y cell line within a high-throughput format. The differentiation media used in our study was similar to Shipley et al.^16^ with slight modifications (e.g. no db-cAMP in the medium, reduced exposure to RA). As recommended in Encinas et al.^15^ RA was removed from the medium after five days, followed by sequential treatment with BDNF (50 ng/mL) for an additional five days. However, as in Shipley et al.^16^ the media was changed from DMEM to Neurobasal supplemented with KCl (20 mM) and B27 (1 ×) and BDNF. The sequential treatment of RA with BDNF employed in our study has been reported to produce a neuronal population with a cholinergic phenotype^14,20,26,27^. However, our protocol can be easily adapted to produce cells of an adrenergic or dopaminergic phenotype, by modifying the media supplements accordingly^20^. It is recommended that future studies employing this protocol to confirm the phenotype of the differentiated cell population using immunocytochemistry for specific markers or other approaches. The additional passaging steps outlined by Shipley et al.^16^ were excluded from our protocol to ensure that each experiment within the 96-well plate started with a known number of cells, therefore maximising experimental reproducibility.

**Figure 1:**
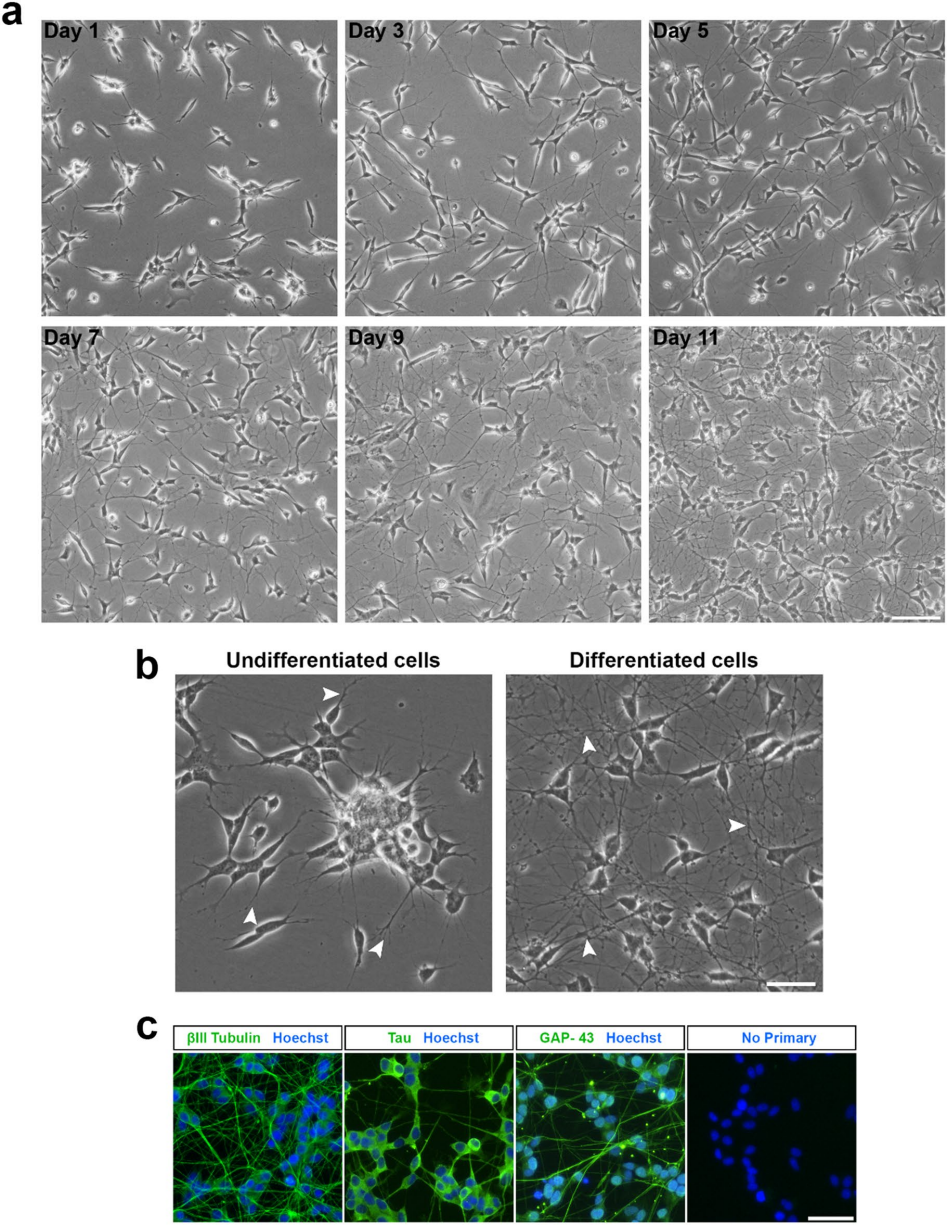
Differentiation of the SH-SY5Y cell line into a neuronal phenotype. (**a**) Phase contrast images of SH-SY5Y cells at various stages of the differentiation process. Cells were treated with RA (10 µM) for five days, followed by BDNF (50 ng/mL) for an additional five days to achieve terminal differentiation by Day 11. Images were acquired using an EVOS FL microscope at ×10 magnification and 43% brightness (scale bar = 50 μm). (**b**) Undifferentiated SH-SY5Y cells possess few short projections and cluster together, while differentiated cells are observed to have many extensive projections. Images were acquired at ×20 magnification and 60% brightness using an EVOS FL microscope (scale bar = 25 μm). βIII-tubulin images were acquired using the GFP filter (excitation 482/25 nm; emission 524/24 nm; exposure 110 ms), while Hoechst nuclei images were acquired using the DAPI filter (excitation 357/44 nm; emission 447/60 nm; exposure 19 ms). White arrowheads point towards neurite extensions. (**c**) Expression of mature neuronal markers by differentiated cells. Fluorescence images were acquired at ×20 magnification using an EVOS FL Auto microscope (scale bar = 25 μm).

The changes in morphology observed in the SH-SY5Y cell population throughout the differentiation period have been presented in Fig. 1a. Within 24 h (day 1), the neurites had visibly elongated compared to the undifferentiated cells. By day 3, the neurite projections appeared more than double in length compared to both the undifferentiated controls (i.e. day 0) and at day 1, with increased branching. These observations are consistent with the literature for this time-point in the differentiation^15,28^. Subsequent removal of the RA, followed by an additional five days incubation with stage II medium produced a population of cells with an extensive neuronal network, as observed in the day 11 image. RA is a vitamin A derivative and a commonly used agent for differentiation induction. Treatment with RA causes cells to reach a post-mitotic state, increase neurite outgrowth and up-regulate the neurotrophic Trk receptors. Moreover, sequential treatment with BDNF has been proven to augment the effects of RA and produce a population of neurotrophin dependent cells^15^.

Phase contrast images in Fig. 1b demonstrate the morphological differences between undifferentiated and differentiated SH-SY5Y cells. Undifferentiated cells aggregated in clusters, with a few, short processes extending outwards from the non-polar cell (white arrowheads), consistent with observations in the literature. These cells are highly proliferative, continuing to increase in density over time and requiring frequent passaging (every 2–3 days). In contrast, differentiated neurons were more evenly distributed on the culture substrate, with many long neurites (white arrowheads) extending to form an interconnected neuronal network. The post-mitotic state induced by differentiation produced a stable population of cells, without any obvious increase in cell density over time. The data also illustrates differences in the shape of the cell body between the undifferentiated and differentiated cells, with the latter noticeably rounder in appearance.

A number of studies have differentiated the SH-SY5Y cell line with RA for a short duration of 2–4 days, at which point the cells are then treated with a chemical factor, to evaluate the inhibitory or stimulatory effect on neurite outgrowth over a specified period^13,29,30^. However, such short differentiation protocols would be insufficient to produce a population of terminally differentiated cells^31^. Hence, their use is unlikely to capture the behaviour of mature neurons^28^. In our protocol, cells were differentiated for a duration of 10 days, similar to Encinas et al.^15^. This duration has previously been reported as sufficient to produce fully differentiated neurons^15^. Immunocytochemical analysis was performed using antibodies against select proteins to confirm the differentiation of the SH-SY5Y cells into a neuronal phenotype. The differentiated cells expressed neuronal-specific markers β-III tubulin, Tau and GAP-43 (Fig. 1c) in agreement with the expression of neuronal markers following RA treatment^18,32^. This confirms that we have successfully differentiated the cells into a neuronal phenotype using our modified protocol. Despite immunocytochemistry and microscopic observations suggesting a culture of high purity, one potential limitation of this study is that the purity of neurons was not specifically quantified.

### Room temperature pre-incubation improves cell plating homogeneity within wells

**Figure 2:**
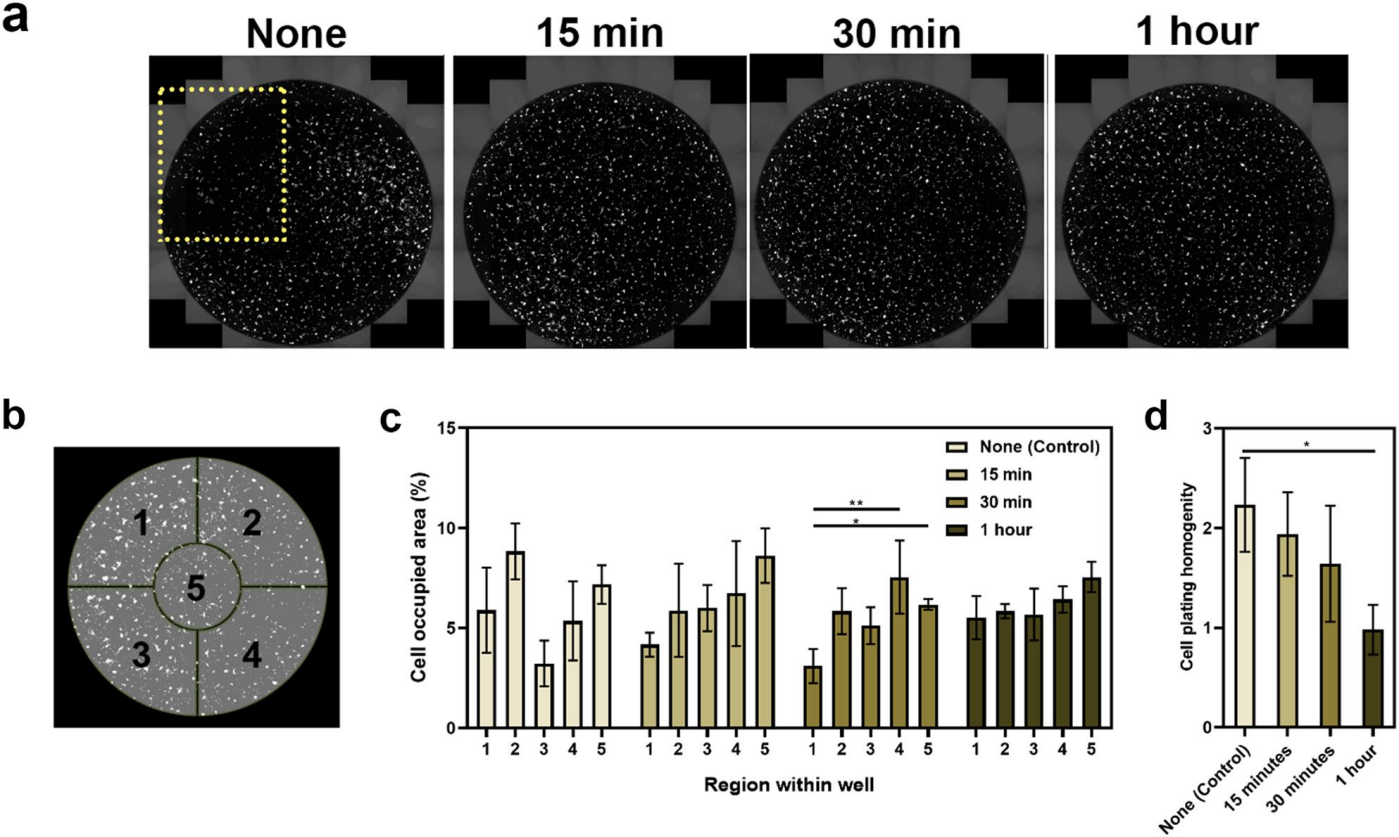
Different room temperature pre-incubation periods affect cell plating homogeneity across the well in 96-well plates. (**a**) Full well scan acquired using the full-well scan function on the EVOS FL Auto microscope with a ×10 objective lens. Yellow box indicates the region where cells are sparsely distributed. (**b**) Schematic illustration of well divisions into five regions for analysis. (**c**) Cell plating homogeneity was determined as the standard deviation between each region. This was used to evaluate the efficacy of pre-incubation for different time periods. Each bar represents the mean (n = 3) ± SD. (**d**) Cell plating homogeneity defined as the standard deviation between each region was used to evaluate the efficacy of pre-incubation for different time periods. Each bar represents the mean (n = 5) ± SD. A one-way ANOVA test and Tukey’s *post-hoc* analysis was performed to evaluate statistical significance where **p < 0.01 and *p < 0.05.

One of the challenges with 96-well plates is achieving an even distribution of the cells throughout the entire well, and minimising any aggregation towards the outside edges. Uneven distribution results in regions of both high cell density and sparse distribution within the same well, which can affect cell behaviour (e.g. the ability to differentiate), confound results in high-throughput screening assays, and impact the reproducibility of experiments between plates and between individual experimenters. Moreover, for assays of neurite outgrowth, uneven distribution can complicate analyses, particularly if the cells are concentrated near the outer edges where the image cannot be accurately focused. For these reasons, it was essential for us to optimise a cell culture technique that could reproducibly achieve uniform plating homogeneity, as part of using differentiated SH-SY5Y cells in a high-throughput assay format. Pre-incubation of the 96-well plates in the Class II biological safety hood for 1 h, prior to transfer into the incubator improved the uniformity of cell distribution within individual wells, with minimised aggregation around well circumference (Fig. 2a). Our findings are consistent with Lundholt et al.^21^, who documented this approach using the CHO-hIR cell line, stating that the efficacy of this technique can vary between different cell lines. In this paper, we are the first to document the value of room temperature pre-incubation for the SH-SY5Y cells.

Plating homogeneity was calculated as the standard deviation of the percentage cell occupied area across five different regions of the well as identified in Fig. 2b. A lower standard deviation was indicative of better plating homogeneity, as there was less variation in percentage cell occupied area between the different regions (Fig. 2c and d). A statistically significant difference in homogeneity was observed with 1 h of pre-incubation (0.982 ± 0.25) compared to no pre-incubation (2.234 ± 0.470) with a *p*-value of 0.037. Although an overall trend of increasing homogeneity was observed as the pre-incubation time increased from 0 to 30 min, these results were not statistically significant. This suggests a minimum period of 1 h is necessary to allow the cells to completely settle and attach. In fact, Lundholt et al. investigated time points of 60-, 90- and 120-min, with 60 min (i.e. 1 h) being the shortest pre-incubation period^21^.

Our experiments demonstrate that a pre-incubation period of 1 h provides cells with sufficient time to settle to the bottom of the well and attach to the substrate. Hence, subsequent movement of the plate between the tissue culture hood and the incubator is less likely to disrupt the position of the cells and the homogeneity is improved. As a tumorigenic cell line, undifferentiated SH-SY5Y cells are considerably robust and this is likely why they were able to withstand suboptimal temperature conditions (i.e. < 37 °C/5% CO_2_) for this duration. Immediate transfer of cells into the incubator upon pipetting the cell suspension (i.e. no pre-incubation), resulted in greater cell density near the outer walls of the well (Fig. 2c). This is likely due to the movement of the suspension disrupting the homogeneity. As uneven cell distribution can affect cell behaviour, achieving uniform plating homogeneity within individual wells is important for a range of high-throughput screening assays not limited to neurite outgrowth (e.g. neurotoxicity studies, cell viability assays).

### Optimising cell plating density for differentiation of SH-SY5Y cells in 96-well plates

Low-density neuronal cultures are known to be more compatible for assays of neurite outgrowth to visualise the effects of chemical factors on neuronal morphology and the complexity of the network formed^2,9^. However, it is important to consider that the cell density can affect the maturation of the neuronal network and synapse formation^33^. Additionally, with a density too sparse it may be challenging to maintain healthy differentiated neuron-like cells in serum-free media, due to reduced paracrine trophic support^34^. Undifferentiated SH-SY5Y cells are highly proliferative. Although induction of differentiation causes the cells to withdraw from the cell cycle and enter a post-mitotic state, this does not occur immediately upon exposure to the differentiation reagents. Rather, the proliferation slows progressively over time until a terminally differentiated state has been attained^15^. Hence, in developing this protocol we sought to identify an initial plating density that would produce a final population of differentiated cells, which are viable in culture and amenable to neurite segmentation and image analyses.

**Figure 3:**
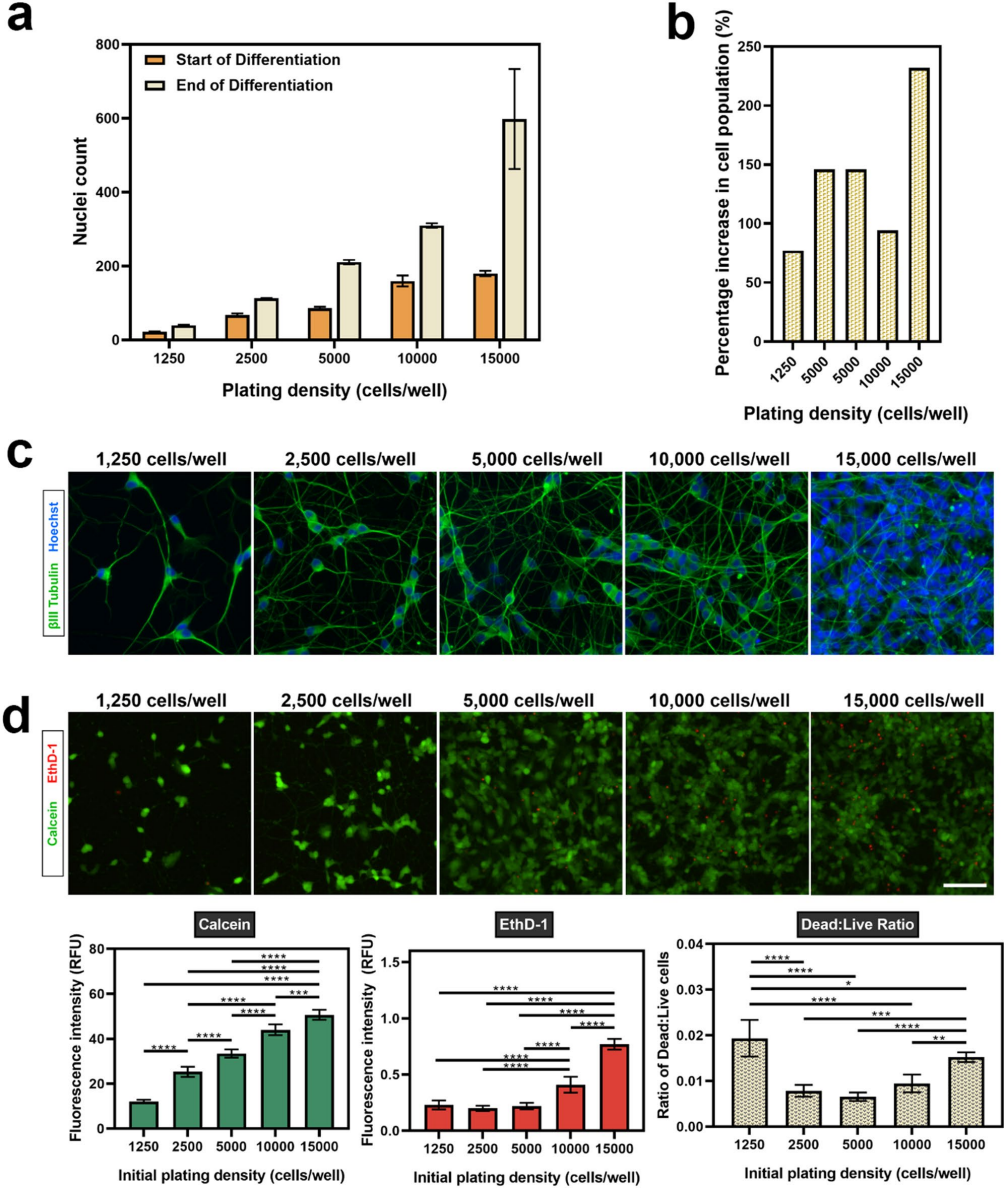
Cell density of plated SH-SY5Y cells increases during the differentiation period. (**a**) The nuclei was counted in four regions across each well at day 1 (start) and day 11 (end) of differentiation. (**b**) Percentage increase in cell population was derived from the nuclei count at day 1 (start) and day 11 (end) of differentiation. Each data point represents the mean (n = 3) ± SD. (**c**) Representative images of terminally differentiated SH-SY5Y cells at varying initial plating densities. As cells proliferate in culture, initial plating densities impact neurite analysis after terminal differentiation. (**d**) The viability of terminally differentiated SH-SY5Y cells was evaluated using a LIVE/DEAD assay and fluorescence intensity measurements used to determine viability. Images were acquired using an EVOS FL Auto microscope at ×20 magnification (scale bar = 25 µm). βIII-tubulin images were acquired using the GFP filter (excitation 482/25 nm; emission 524/24 nm; exposure 110 ms), while Hoechst nuclei images were acquired using the DAPI filter (excitation 357/44 nm; emission 447/60 nm; exposure 19 ms). Each bar represents the mean (n = 5) ± SD. A one-way ANOVA test and Tukey’s post-hoc analysis was performed to evaluate statistical significance where ****p < 0.0001, ***p < 0.001, **p < 0.01 and *p < 0.05.

Figure 3a and Supplementary Fig. S4 illustrate the proliferative nature of the SH-SY5Y cells throughout the 10 day differentiation period, confirming a difference between the initial plating density and final number of cells within each well. Nuclei were counted in select regions across the extent of the well at day 1 of the differentiation protocol (i.e. the start of differentiation), and on a separate plate that had been differentiated until day 11 (i.e. the end of differentiation). The highest percentage increase in cell population (~ 232%) was for cells plated at an initial density of 15,000 cells/well, while the lowest percentage increase in cell population were noted for cells plated at 2,500 cells/well (~ 67%) and 1,250 cells/well (~ 77%) (Fig. 3b). These observations were expected, as undifferentiated SH-SY5Y cells are highly proliferative in culture, resulting in the increase in cell density from the day of initial plating. Hence, it was necessary to optimise the initial plating density of the undifferentiated cells to permit neurite extension and visualisation over a 10 day culture period.

These experiments provided further insight around extent of this proliferation, helping to inform the experimental design for subsequent neurite outgrowth assays within a high-throughput 96-well plate format. In Fig. 3c, the impact of initial plating density on the visualisation of individual neurites labelled with βIII-tubulin has been presented. It was important to determine an initial plating density that would result in a final population of differentiated cells with neurites that could be distinguished and are amenable to identification and segmentation during automated image analysis. Neurite length, extent of branching (e.g. number of branch points, number of end points), cell body size and cell body shape are all quantifiable parameters of neuronal morphology that can provide interesting information regarding the effect of an inhibitory or stimulatory treatment. However, the ability to accurately extract this information is complex and largely sensitive to the density of the neuronal network amongst other factors (e.g. quality of ICC staining, magnification and resolution of images, signal to background ratio). This is further complicated by the fact that neurites are fine, unevenly shaped structures.

Cells initially plated at a density of 15,000 cells/well had become too overcrowded by the end of the differentiation to successfully discern individual neurites (Fig. 3c). The clustering of the nuclei illustrates the reduced surface area available for the individual cells to extend projections, and it would be challenging to image and analyse the effect of a chemical stimulus on neurite outgrowth at this density. Moreover, at such a high density there would be more rapid depletion of nutrients from the media, and a lower viability as confirmed by the results of the LIVE/DEAD assay presented in Fig. 3d. In this figure, the fluorescence intensity of calcein and EthD-1 correlate to the relative number of live and dead cells in the culture, respectively.

In our study, we reported the ratio of dead to live cells as an indicator of viability at each of the plating densities, with a higher ratio suggestive of lower viability. At an initial plating density of 15,000 cells/well, the ratio of dead to live cells (0.015 ± 0.0011) was approximately twice as great than that at 2,500 cells/well (0.008 ± 0.0013) and two and a half times greater than at 5,000 cells/well (0.007 ± 0.0009). Though the density had substantially reduced at 10,000 cells/well, the clustering of nuclei was still seen to be present in the culture, and addition of a growth-promoting factor (e.g. neurotrophin) at this density would likely result in additional neurite outgrowth that would be difficult to segment. In contrast, at the lowest density of 1,250 cells/well, the individual neurites were clearly distinguishable. While such a density would be suited for analysis of neurite outgrowth at a single cell level, the ratio of dead to live cells (0.019 ± 0.004) was the highest. Additionally, the cells were sparsely distributed across the well, which would affect cell–cell signalling^34^.

From these results, an initial plating density of 2,500 cells/well was selected as the optimal density to differentiate SH-SY5Y cells in a 96-well plate for neurite outgrowth assays. At this density, the ratio of dead to live cells was comparatively low. Importantly, this density permitted clear distinction of individual neurites that could be traced back to the original cell body.

### Comparison of different cell detachment reagents for re-plating differentiated SH-SY5Y cells

Next, we investigated whether the SH-SY5Y cells could be re-plated after differentiation within the high-throughput 96-well plate format. The ability to re-plate differentiated cells after they have extended neurite projects within a complex network enables the study of neurite regeneration and synapse assembly in cells that are post-mitotic, as the neurites are initially damaged during re-plating^19^. This could be a relevant model for researchers investigating neurite regeneration following traumatic injury (e.g. spinal cord injury), to identify potential growth-promoting molecules and screening therapeutic doses. Within the existing literature, the SH-SY5Y cells have typically been differentiated on the same substrate in which the final assay would be performed. To the best of our knowledge, this is the first in the literature to compare these widely available detachment reagents for re-plating differentiated SH-SY5Y cells and report these findings.

Differentiated SH-SY5Y cells detached using trypsin–EDTA (0.05%), Versene and GCDR could be successfully re-plated as a dissociated neuronal culture, with projections beginning to re-extend by 24 h (Supplementary Fig. S5). When selecting the reagents to compare for these experiments, it was important for us to consider that neuronal cells are sensitive in culture and susceptible to structural damage. Hence, we investigated several reagents which would be typically used for passaging stem cells (e.g. Accutase, Dispase), and a low concentration solution of the harsher serine protease enzyme, trypsin–EDTA. Moreover, all the reagents investigated are commonly used for cell culture. In fact, Accutase has previously been reported to successfully detach human iPSC-derived neurons after differentiation^19^. It was interesting to observe that in our study, Accutase and Dispase both led to the formation of irregularly shaped free-floating clusters of cells, whilst trypsin–EDTA (0.05%), Versene and GCDR produced dissociated cultures (Supplementary Fig. S5).

**Figure 4:**
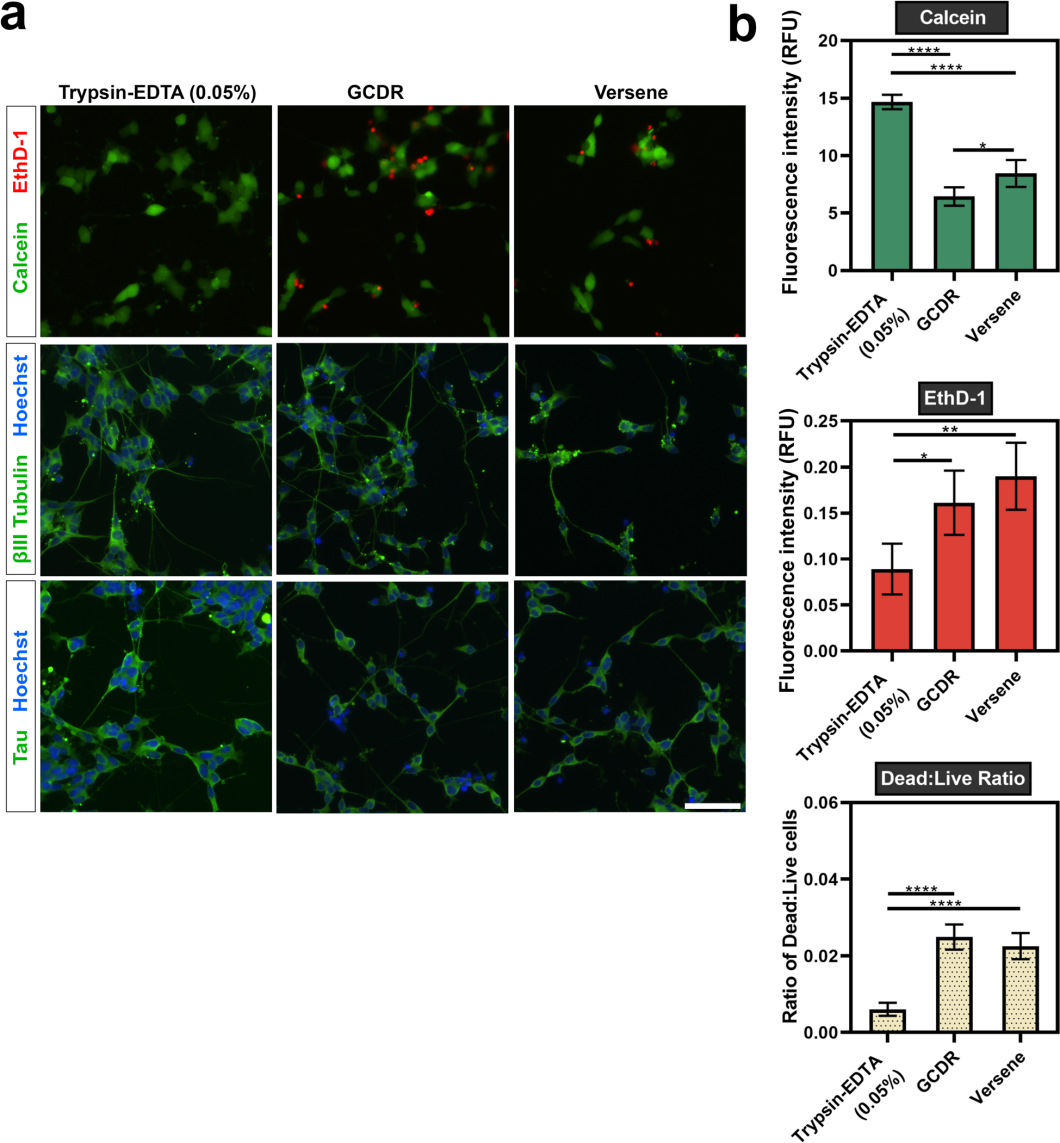
Detachment and re-plating of differentiated SH-SY5Y cells. Terminally differentiated SH-SY5Y cells were re-plated using trypsin–EDTA (0.05%), GCDR or versene and assessed for viability and expression of neuronal markers 48 h after re-plating. (**a**) Microscopic images of calcein (live) and EthD-1 (dead) labelled cells. Images were acquired using an EVOS FL Auto microscope with a ×20 objective lens (scale bar = 100 µm). βIII-tubulin images were acquired using the GFP filter (excitation 482/25 nm; emission 524/24 nm; exposure 110 ms), while Hoechst nuclei images were acquired using the DAPI filter (excitation 357/44 nm; emission 447/60 nm; exposure 19 ms). (**b**) Fluorescence emission of calcein and EthD-1 was measured to evaluate viability after re-plating. A one-way ANOVA test and Tukey’s post-hoc analysis was performed to evaluate statistical significance where ****p < 0.0001, ***p < 0.001, **p < 0.01 and *p < 0.05.

Immunocytochemistry confirmed the expression of neuronal markers βIII-tubulin and tau following detachment and re-plating with trypsin–EDTA (0.05%), Versene and GCDR (Fig. 4a). The viability of the cells re-plated with each of the different detachments reagents was then evaluated using a LIVE/DEAD assay (Fig. 4a and b). In this paper, our focus was to establish a dissociated single-cell culture model for analysis of neurite outgrowth. Therefore, cells re-plated using Accutase and Dispase were excluded from subsequent experiments, due to the formation of free-floating clusters of cells. As observed in the graphs presented in Fig. 4b [Left], the proportion of live cells, indicated by the calcein fluorescence emission, was around two times greater for cells detached with trypsin–EDTA (0.05%) (14.66 ± 0.62) compared to GCDR (6.44 ± 0.81) and Versene (8.44 ± 1.17). Similarly, trypsin–EDTA (0.05%) had almost half the proportion of dead cells, indicated by the EthD-1 emission, at a value of 0.0891 ± 0.028. For GCDR and Versene, these values were at 0.161 ± 0.035 and 0.190 ± 0.036, respectively. The ratio of dead to live cells in cultures re-plated using trypsin–EDTA (0.006 ± 0.0017) was around four times lower than those detached with GCDR (0.025 ± 0.0033) and Versene (0.023 ± 0.0034).

From the detachment reagents tested, our findings supported the use of trypsin–EDTA (0.05%) as the most effective for differentiated SH-SY5Y cells. Importantly, although the cells remain viable, the re-plating caused damage to the neurite projections—as seen in the before and after images in Supplementary Fig. S5. This would have occurred due to both the detachment reagents and the mechanical trituration prior to re-plating, and similar observations have been reported by Calabrese et al.^19^ following the detachment of iPSC-derived neurons.

### Optimising image acquisition for neurite outgrowth

**Figure 5:**
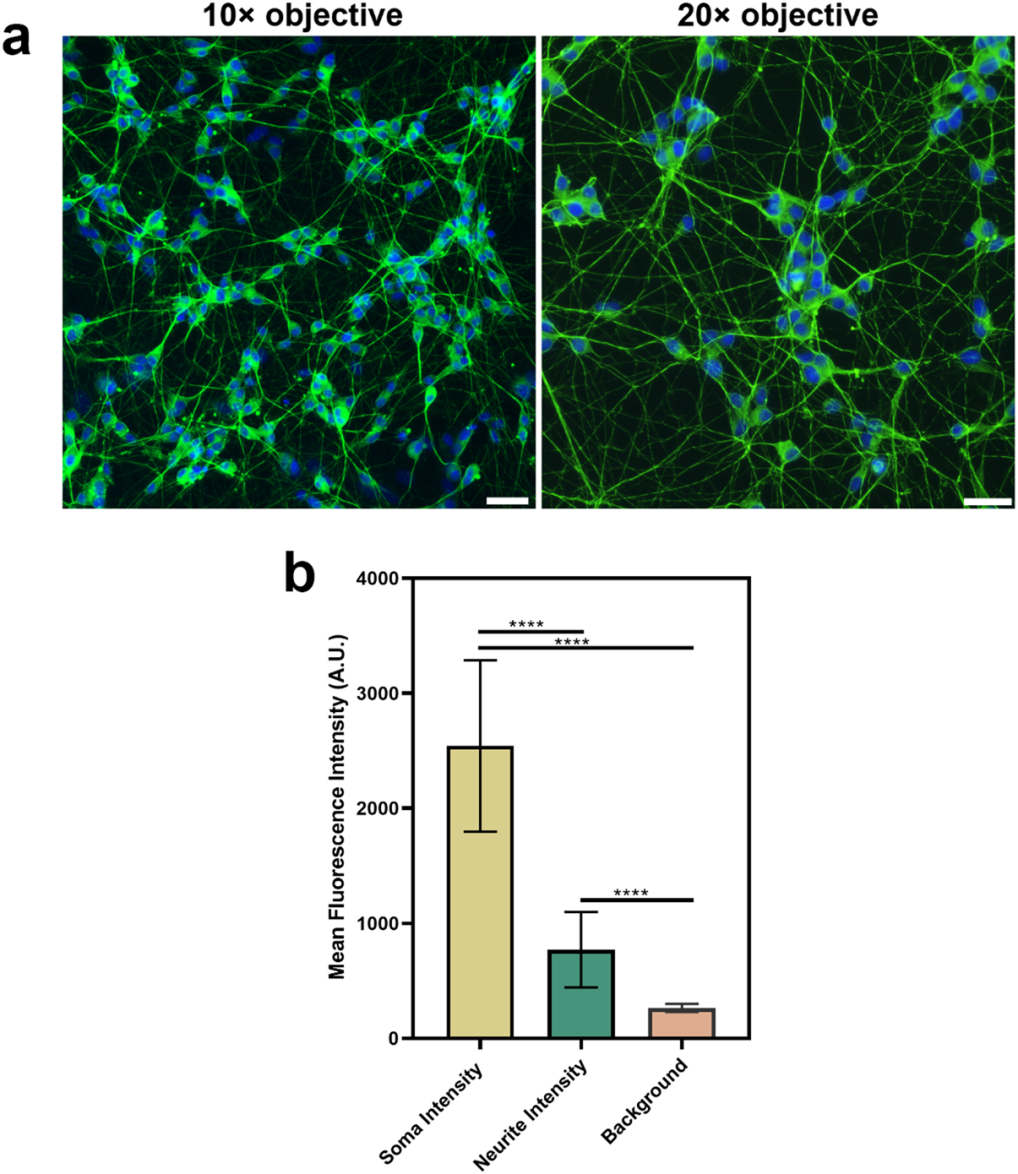
Image acquisition for neurite outgrowth analysis. (**a**) Comparison of images at ×10 (left) and ×20 (right) magnification (scale bars = 25 µm). Differentiated SH-SY5Y cells were labelled with βIII-tubulin and a Hoechst nuclear counterstain. βIII-tubulin images were acquired using the GFP filter (excitation 482/25 nm; emission 524/24 nm), while Hoechst nuclei images were acquired using the DAPI filter (excitation 357/44 nm; emission 447/60 nm). Although the ×10 objective increases the number of cells in the field of view, it is more difficult to visualise finer neurite processes. The ×20 objective acquires images with a higher resolution to distinguish these features. (**b**) Differences in mean fluorescence intensity of βIII tubulin between the soma, neurites and background. Analysis was performed on images acquired using a ×20 objective. Each bar represents the mean (n = 250 points) ± SD. A one-way ANOVA test and Tukey’s post-hoc analysis was performed to evaluate statistical significance where ****p < 0.0001.

To visualise neurite outgrowth, neurotrophin-treated cells and the controls were labelled with βIII-tubulin, a commonly used marker for measuring neurite outgrowth, in combination with a Hoechst nuclear stain^9^. In preliminary experiments, a 10 × objective and 20 × objective were both trialled for image acquisition (Fig. 5a). However, while the 10 × objective allowed a greater number of cells to be visualised in the field of view, the 20 × objective was superior in terms of neurite visualisation and image resolution. This has been described by Sirenko et al.^9^, where the use of a 20 × objective enhanced detection of the finer neuronal processes within the image. Therefore, in our experiments we acquired three images at 20 × objective were acquired for each well of the 96-well plate within the central region, to avoid difficulties in focusing with the outer walls.

As seen in Fig. 5b, the mean fluorescence intensity of βIII-tubulin in the neuronal soma (2542 ± 743) is around three times greater than the intensity in the neurites (769 ± 327) with a *p*-value < 0.0001. This is important to ensure accurate detection and segmentation of the soma during analysis^25^. There is considerable variation in the intensity of the neurites, as indicated by the standard deviation, which was almost half the value of the mean. Given the density of the SH-SY5Y neuronal network (Fig. 5a), this observation was as expected and is consistent with the literature^25^. Pani et al. state that in a dense neurite network there are regions with high intensity amongst thinner, faintly stained neuritis—particularly further away from the soma^25^. In fact, there are neurites with weaker fluorescence intensity signals having intensity values that are higher than, yet still comparatively close to the mean background intensity (264 ± 35). Taken together, this suggests the importance of acquiring an image of a quality that is compatible with automated image analyses. The neurite network image analysis script MorphoNeuroNet^25^ compatible for use with FIJI^22^ was selected for used in our study, as it has been reported as compatible with cultures that have formed a neuronal network over a period of 10 days. Additionally, Pani et al.^25^ have demonstrated the ability of this script to detect neurites weak fluorescence intensity. As such, it was more appropriate for this study compared to other available image analysis plugins. As the protocols outlined in this paper have optimised the universal steps differentiation of the SH-SY5Y cell line within a 96-well plate for neurite outgrowth analysis, it would likely be suited for researchers to also use their own image analysis pipelines for quantifying neurite outgrowth.

### High-throughput screening of neurotrophins on differentiated SH-SY5Y cells

Finally, to demonstrate the efficacy of our optimised protocol in use, we aimed to investigate the effects of neurotrophic stimulation of differentiated SH-SY5Y cells in a 96-well plate format under four different conditions. A dose–response assay using NGF, BDNF and NT-3 at concentrations ranging from 12.5 to 400 ng/mL was performed on: (i) terminally differentiated cells (ii) terminally differentiated cells in the presence of soluble CSPG growth inhibitors and (iii) terminally differentiated cells that have been re-plated with trypsin–EDTA (0.05%). As described earlier, terminally differentiated cells were used as a model for mature neurons. Meanwhile, CSPGs are known inhibitors of neurite outgrowth^24^. By conducting a neurotrophin dose–response assay in the presence of a known neurite outgrowth inhibitor, we sought to identify whether a difference could be detected when cells were differentiated using our optimised protocol. Finally, re-plating of terminally differentiated neurons causes damage to the neurite projections, allowing us to investigate the effects of neurotrophins on their re-growth. In our study, the effect on neurite outgrowth has been reported as a ratio of the total neurite length to the number of nuclei, to account for any differences in nuclei number between images.

**Figure 6:**
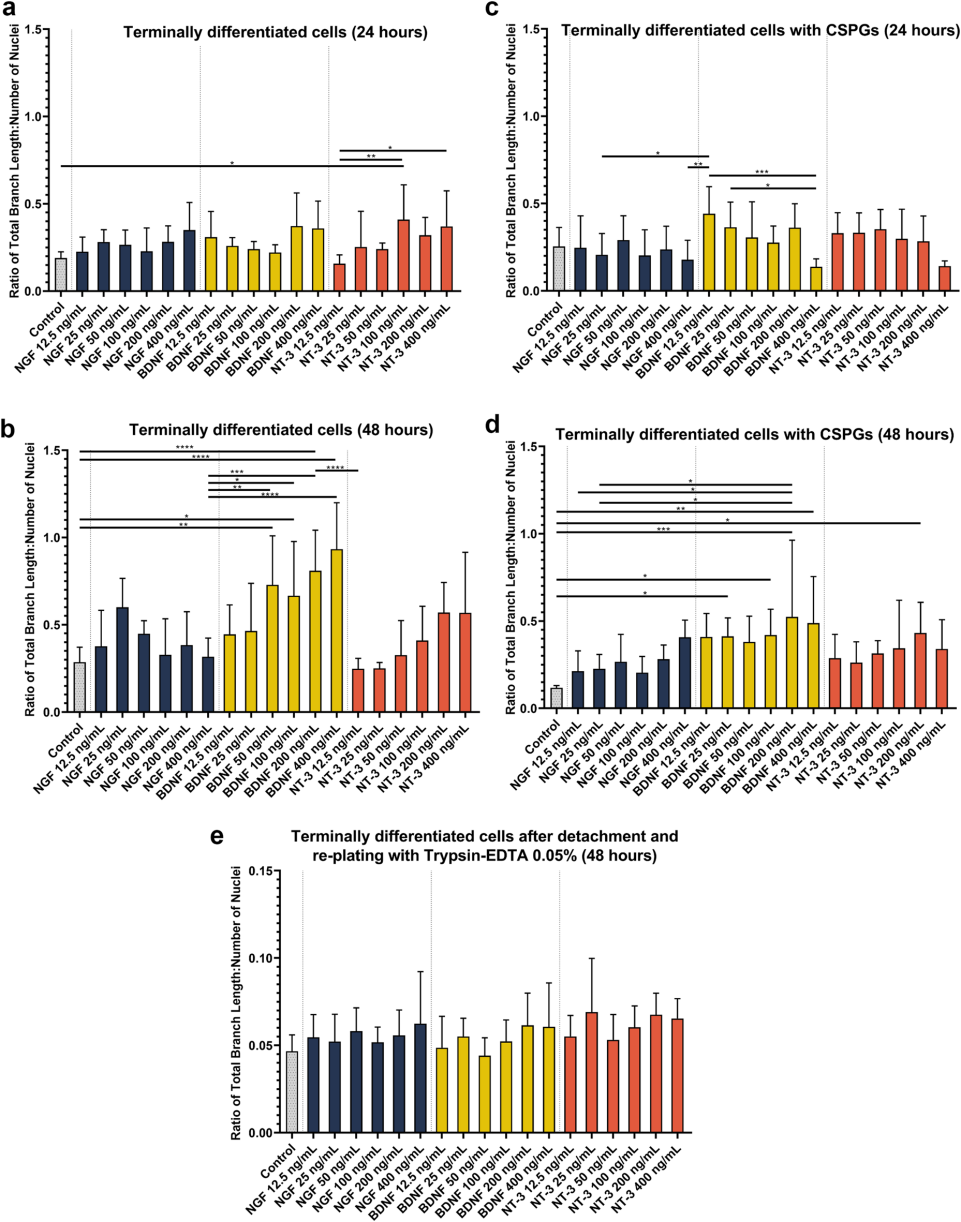
Neurite outgrowth quantification in response to treatment with NGF, BDNF or NT-3. (**a**) Terminally differentiated cells incubated with neurotrophin medium for 24 h and (**b**) Terminally differentiated cells incubated with neurotrophin medium for 48 h. (**c**) Terminally differentiated cells incubated with neurotrophin medium and 10 µg/mL CSPG for 24 h. (**d**) Terminally differentiated cells incubated with neurotrophin medium and 10 µg/mL CSPG for 48 h. (**e**) Terminally differentiated cells re-plated with trypsin–EDTA (0.05%) and incubated with neurotrophin medium and 10 µg/mL CSPG for 48 h. Each bar represents the mean ± SD. A one-way ANOVA test and Tukey’s post-hoc analysis was performed to evaluate statistical significance where ****p < 0.0001, ***p < 0.001, **p < 0.01 and *p < 0.05.

Addition of a chemical stimulus after differentiation of the SH-SY5Y cell line has been previously reported in the literature^13^. In our study, neurotrophic stimulation of terminally differentiated cells (Fig. 6a and b) resulted in statistically significant changes in outgrowth between NT-3 (100 ng/mL) and the control (*p* = 0.0295) after 24 h, and between both NT-3 (400 ng/mL) and NT-3 (100 ng/mL) compared to NT-3 (12.5 ng/mL) with *p*-values of 0.0453 and 0.0045, respectively. Interestingly, after 48 h a far more drastic increase in neurite outgrowth was observed, particularly for cells treated with BDNF. The images of the cells at 48 h have been presented in Supplementary Fig. S6. Compared to the control, BDNF 200 ng/mL and 400 ng/mL increased outgrowth by approximately three times compared to the control (*p-*value =  < 0.0001), while BDNF 100 ng/mL and BDNF 50 ng/mL increased outgrowth by approximately two and a half times. As stated earlier, RA induces the expression of TrkB but not TrkA^35,36^. Further, TrkB is predominantly a receptor for BDNF but can also bind NT-3 to a lesser degree^35^. This supports our observations that BDNF was the more robust stimulator of outgrowth of the neurotrophins investigated in this study, with some response seen in NT-3 treated cells.

We then investigated the effects of neurotrophic stimulation in the presence of soluble CSPGs (10 µg/mL) (Fig. 6c and d). At 24 h, no statistically significant difference was observed between neurotrophin-treated cells and the control. At 48 h, BDNF treated cells exhibited the highest outgrowth compared to the control, NGF, and NT-3 treated cells. Moreover, the overall outgrowth of BDNF treated cells was lower in the presence of soluble CSPGs compared to BDNF alone. At 50 ng/mL and 100 ng/mL of BDNF the outgrowth in terminally differentiated cells without CSPGs was 0.729 and 0.666, respectively. This was reduced to 0.380 and 0.420 in the presence of soluble CSPGs. These findings show that our protocol can also be used to measure the effect of molecules that inhibit neurite outgrowth as well as study molecules that have the potential to overcome this inhibition. Images of the cells at 48 h are presented in Supplementary Fig. S7.

Finally, no significant neurite outgrowth was seen in the re-plated terminally differentiated cells in response to neurotrophic stimulation (Fig. 6e). Images of the cells are presented in Supplementary Fig. S8. One reason for this is that with the damage to the neurite projections during the re-plating procedure, the existing neurotrophin receptors may have also been affected. Hence, the duration for which the cells were exposed to the chemical stimulus (48 h) in our study may not have been sufficiently long enough to elicit a detectable response in cells that have undergone a re-plating procedure.

This study has several strengths compared to existing assays. SH-SY5Y cells have commonly been used for neurite outgrowth in an undifferentiated state, or during differentiation^18,30,37^. Here, we have demonstrated the applicability of terminally differentiated SH-SY5Y cells and re-plated differentiated cells to be used as a post-mitotic cell model for measuring neurite outgrowth in response to chemical stimulation. Additionally, re-plated differentiated cells can also enable study of axonal regeneration and synapse re-assembly^19^. Our study also details an optimised initial seeding density where the density of neurites are amenable to analysis of total branch length at the end of differentiation. However, there are also some limitations of our study. Firstly, immunocytochemistry using βIII-tubulin was used to label neurites for outgrowth quantification. Although this is a commonly used approach, a number of time-consuming manual processing steps are required which can be made further challenging in a 96-well plate format. It would be valuable for future studies explore alternative techniques to visualise neurites such as GFP transfection of cells^38^, calcein staining^9^ or a neurite outgrowth staining kit^39^ which are faster to use, and in which the cells can remain alive. This would enable researchers to also simultaneously stain for dead cells, and evaluate viability in response to the different chemical agents. Moreover, in the current study, an EVOS FL Auto microscope was used for image acquisition. To further facilitate high-throughput screening, it would be valuable for future studies to employ a high-content image acquisition and analysis system^38,40^.

## Conclusions

In this paper, we systematically optimised a differentiation protocol for the SH-SY5Y cell line within a 96-well plate format, and demonstrated its effectiveness as an assay for measuring neurite outgrowth. The described techniques can also be applied to differentiate SH-SY5Y cells in a high-throughput format for other screening assays such as neurotoxicity and viability studies. Pre-incubation of the plates for 1 h at room temperature prior to movement into the incubator improved the plating homogeneity of the cells across the well. This is an important step in high-throughput screening assays, as regions of varied cell distribution can influence cell behaviour and experimental reproducibility. An initial plating density of 2,500 cells/well was identified as appropriate to balance the low-density required for neurite visualisation and image analysis without influencing the viability of the cells. Terminally differentiated cells were detached and re-plated using the detachment reagents trypsin–EDTA (0.05%), Versene and GCDR to form a single cell culture. Accutase and Dispase led to the formation of a free-floating cluster of cells. Finally, high-throughput neurite outgrowth assays investigating the effects of NGF, BDNF and NT-3 on differentiated SH-SY5Y cells were used to validate our protocol. Our observations indicate that for the neurotrophins tested, BDNF promoted more significant outgrowth in the differentiated SH-SY5Y cells in the presence and absence of soluble neurite outgrowth inhibitors. This demonstrates the efficacy of our protocol for an initial screening across multiple chemical stimulants and concentrations. To then fully elucidate the mechanisms behind the observations and obtain more detailed concentration-dependent information, future experiments would involve analysing the effects of neurotrophic stimulation at a single-cell level. The presented protocol achieves fully differentiated and homogenously distributed SH-SY5Y cells in a 96-well plate format, useful for researchers using these cells in high-throughput screening assays.

## Supplementary Information

Supplementary Information.

## Data Availability

The datasets generated and/or analysed during the current study are available from the corresponding author on reasonable request.
