# Supplementary material for: Optimised techniques for high-throughput screening of differentiated SH-SY5Y cells and application for neurite outgrowth assays
Source: Sci Rep. 2021 Dec 14;11:23935. doi: 10.1038/s41598-021-03442-1 (PMC8671469; doi:10.1038/s41598-021-03442-1)
Supplement: Supplementary file 1 — Supplementary Information. [file 41598_2021_3442_MOESM1_ESM.pdf]

Supplementary information

Optimised techniques for high-throughput screening of differentiated SH-SY5Y cells and application for neurite outgrowth assays

Anusha Dravid[1], Brad Raos[1], Darren Svirskis[1], Simon J O'Carroll[2*]

[1] School of Pharmacy, Faculty of Medical and Health Sciences, University of Auckland, Private Bag 92019, Auckland, New Zealand
[2] Department of Anatomy and Medical Imaging, School of Medical Sciences, Faculty of Medical and Health Sciences, University of Auckland, Private Bag 92019, Auckland, New Zealand

***Corresponding Author:**

Dr Simon J O'Carroll

Department of Anatomy and Medical Imaging, School of Medical Sciences. Faculty of Medical and Health Sciences, University of Auckland, Private Bag 92019, Auckland, New Zealand

Email: s.ocarroll@auckland.ac.nz

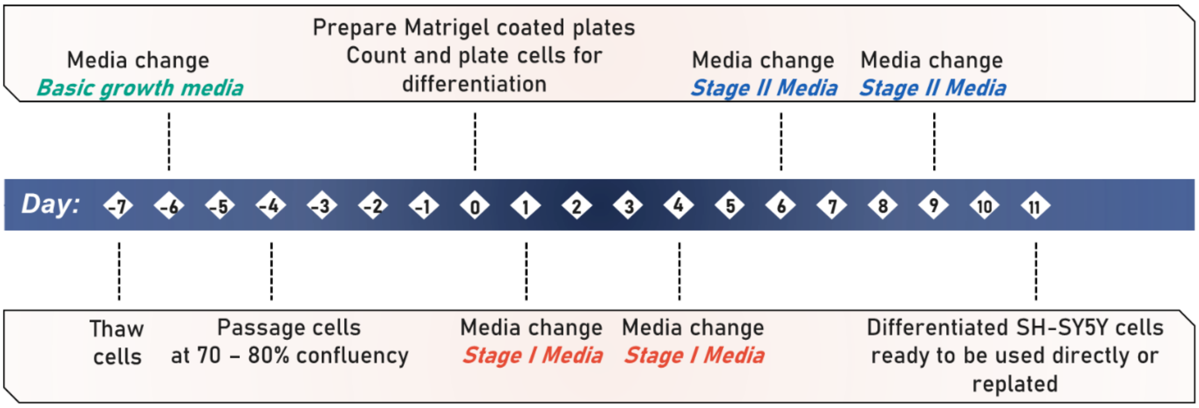

Supplementary Figure S1. Timeline and protocol for SH-SY5Y cell differentiation. A frozen cryovial of undifferentiated SH-SY5Y cells was thawed, and passaged once before plating for differentiation on Matrigel-coated plates. Cells were incubated for 5 days in Stage I media consisting of RA (10 µM), followed by 5 days in Stage II media consisting of BDNF (50 ng/mL) to produce a population of differentiated neurons.

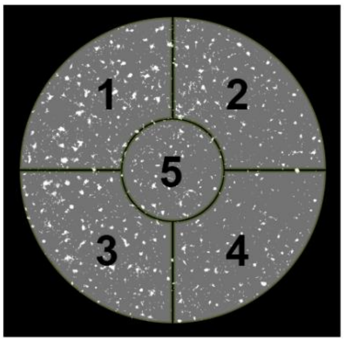

Supplementary Figure S2. Analysis of cell distribution in 96-well plates. A custom ImageJ macro was written to quantify the percentage cell occupied area in five regions across the well. The standard deviation of the mean percentage cell occupied area between each of the regions 1 – 5 was used to evaluate plating homogeneity, with a lower standard deviation corresponding to greater homogeneity.

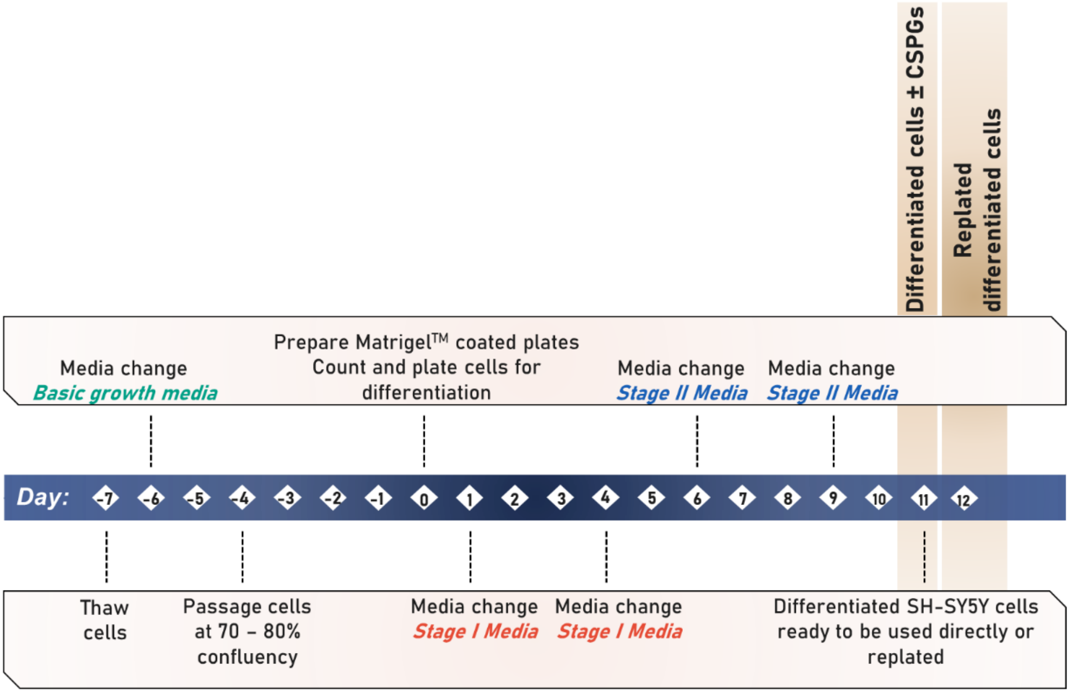

Supplementary Figure S3: Schematic representation of the dose-response assay. The bands on the differentiation timeline indicate when the neurotrophins were added for each of the different stages investigated.

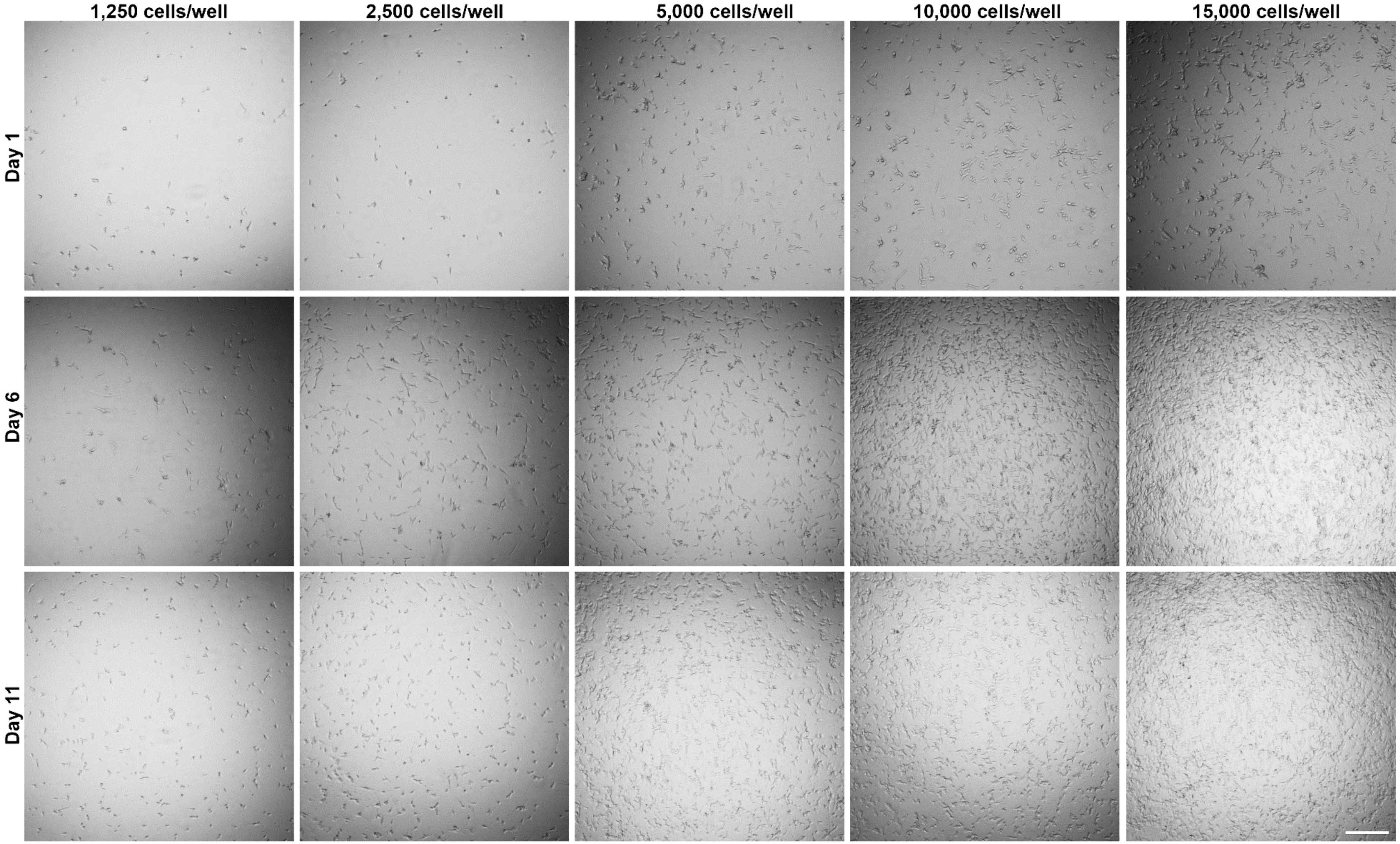

Supplementary Figure S4: Phase contrast images of SH-SY5Y cell proliferation from initial plating density throughout the differentiation period. Undifferentiated SH-SY5Y cells are highly proliferative in culture until sufficiently exposed to differentiation reagents, resulting in an increase in cell density from the initially plated number. Images were acquired using an EVOS FL microscope at 4× magnification (scale bar = 250 µm).

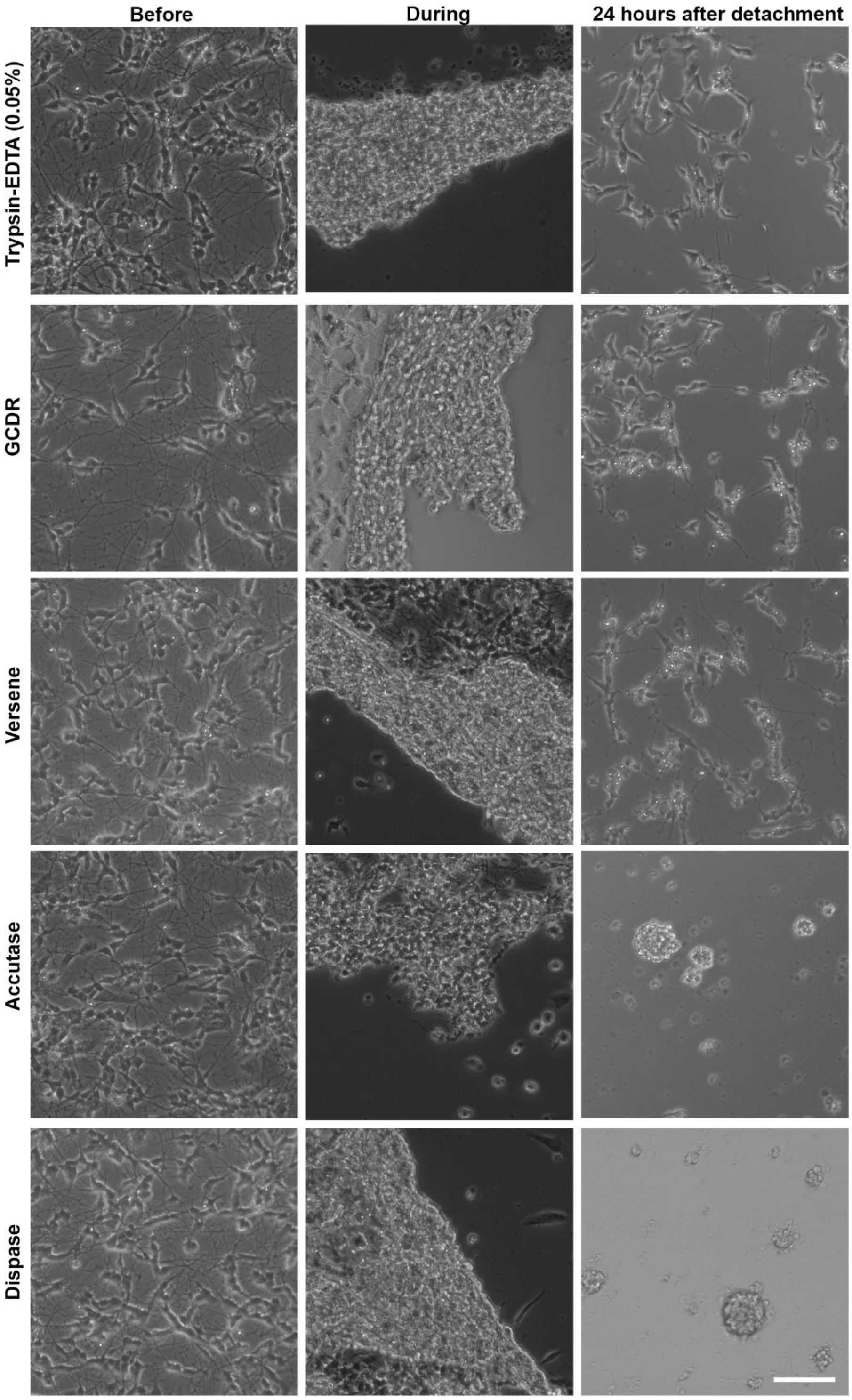

Supplementary Figure S5: Phase contrast images of differentiated SH-SY5Y cells before, during and 24 hours after detachment using either Trypsin-EDTA (0.05%), GCDR, Versene, Accutase or Dispase. Images were acquired using an EVOS FL microscope with a 10× objective (scalebar = 100 µm)

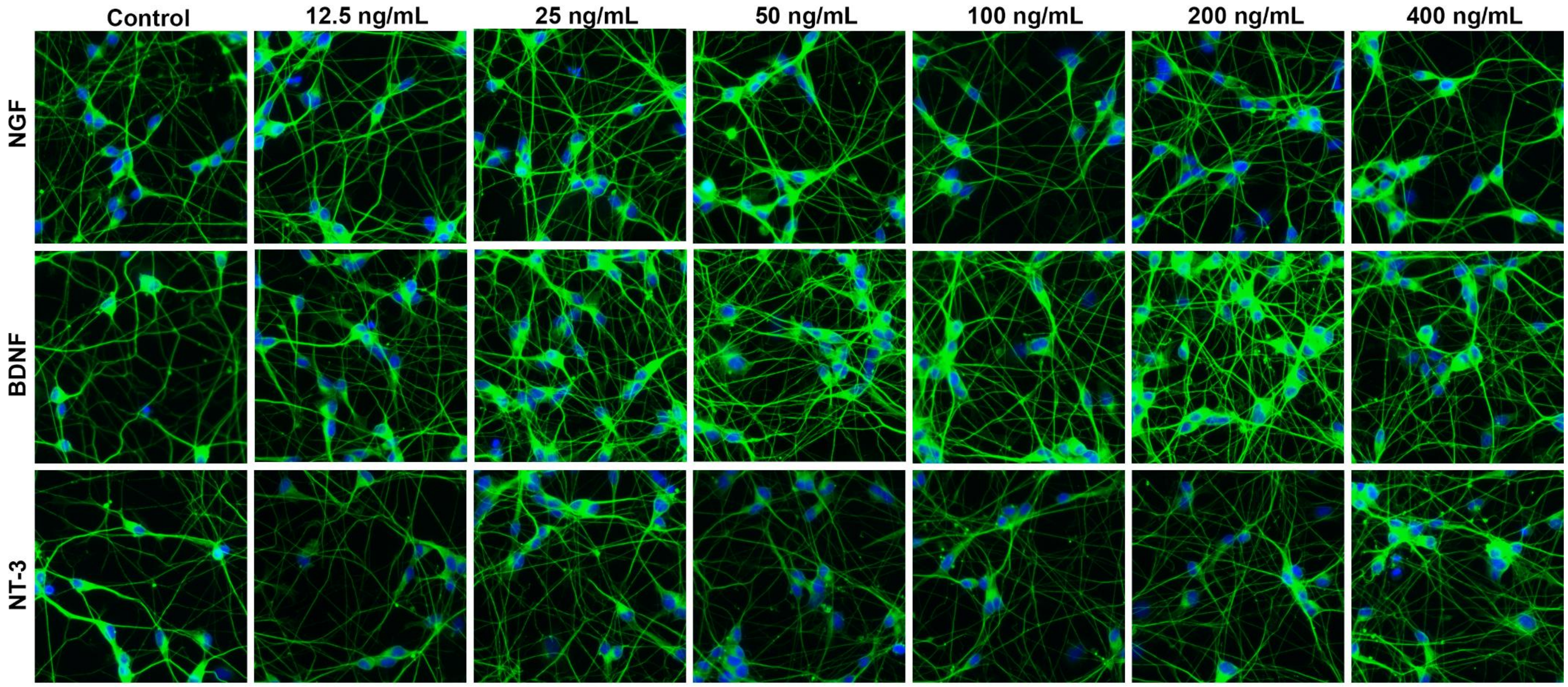

Supplementary Figure S6: Images of terminally differentiated SH-SY5Y neurons after 48 hours of exposure to NGF, BDNF or NT-3. Each neurotrophin was investigated at a concentration of 12.5 ng/mL, 25 ng/mL, 50 ng/mL, 100 ng/mL, 200 ng/mL and 400 ng/mL in a 96-well plate format. Cells were labelled with βIII-tubulin with a Hoechst nuclear counterstain. Images were acquired using the EVOS FL Auto microscope with a 20× objective lens. The 16-bit nuclei and neurite images were acquired in TIF format, and merged to form a single composite image with separate nuclei and neurite channels using FIJI software.

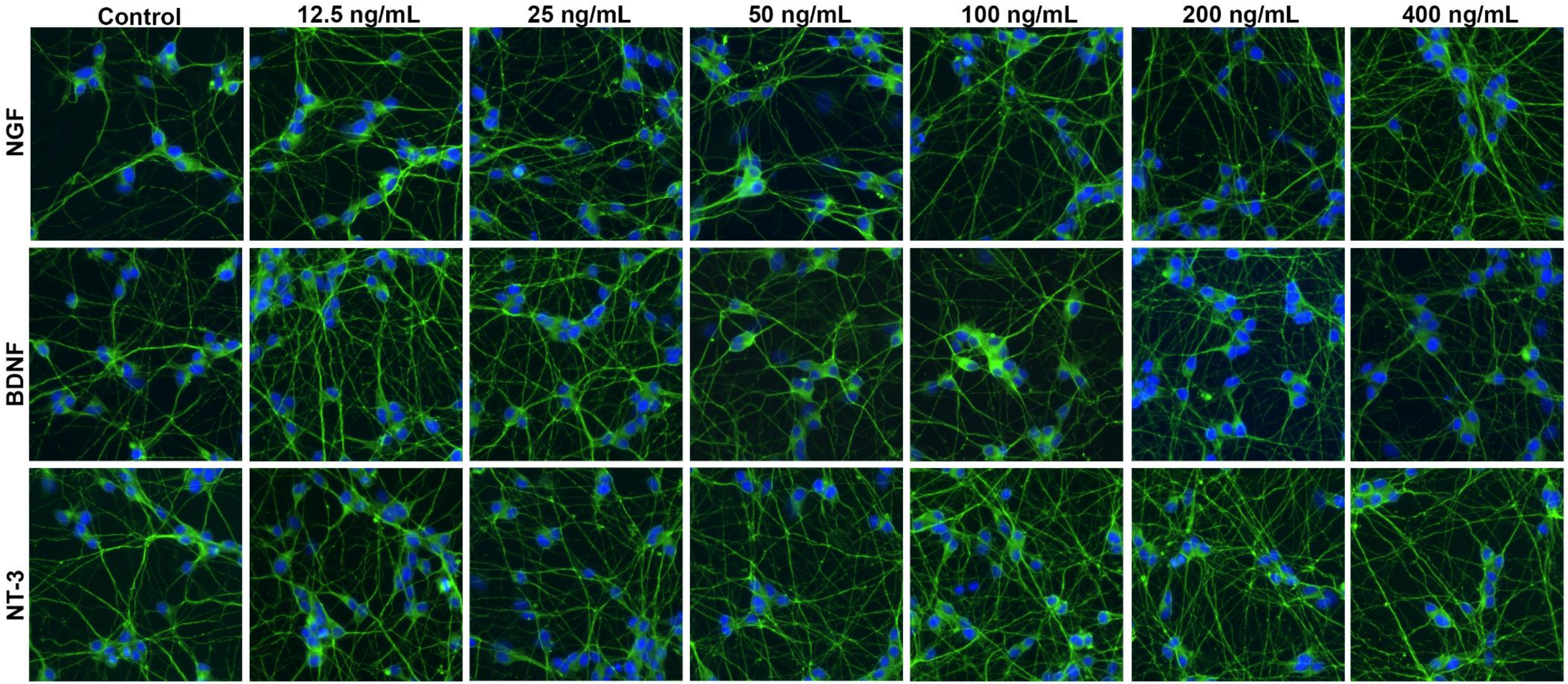

Supplementary Figure S7: Images of terminally differentiated SH-SY5Y neurons after 48 hours of exposure to NGF, BDNF or NT-3 in the presence of 10 µg/mL soluble CSPGs. Each neurotrophin was investigated at a concentration of 12.5 ng/mL, 25 ng/mL, 50 ng/mL, 100 ng/mL, 200 ng/mL and 400 ng/mL in a 96-well plate format. Cells were labelled with βIII-tubulin with a Hoechst nuclear counterstain. Images were acquired using the EVOS FL Auto microscope with a 20× objective lens. The 16-bit nuclei and neurite images were acquired in TIF format, and merged to form a single composite image with separate nuclei and neurite channels using FIJI software.

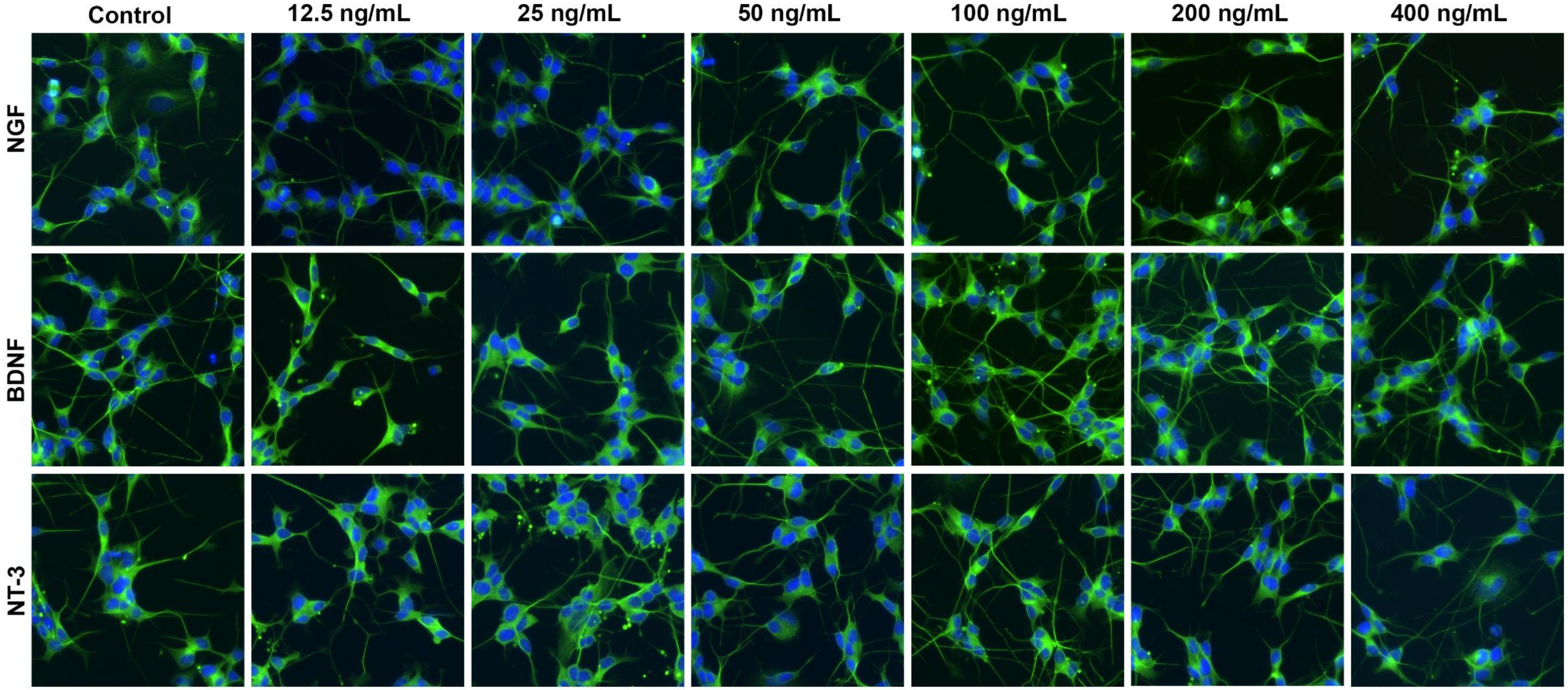

Supplementary Figure S8: Images of terminally differentiated SH-SY5Y neurons, re-plated with Trypsin-EDTA (0.05%) and 48 hours of exposure to NGF, BDNF or NT-3. Each neurotrophin was investigated at a concentration of 12.5 ng/mL, 25 ng/mL, 50 ng/mL, 100 ng/mL, 200 ng/mL and 400 ng/mL in a 96-well plate format. Cells were labelled with βIII-tubulin with a Hoechst nuclear counterstain. Images were acquired using the EVOS FL Auto microscope with a 20× objective lens. The 16-bit nuclei and neurite images were acquired in TIF format, and merged to form a single composite image with separate nuclei and neurite channels using FIJI software.

Supplementary Table S1: Steps for replating differentiated SH-SY5Y neurons using different detachment reagents

Detachment Reagent	Steps
**Trypsin-EDTA (0.05%)**	Trypsin-EDTA (0.05%) was warmed in a 37°C water bath prior to use. Old culture media was aspirated and discarded from each well. Cells were rinsed briefly with 1× PBS. Cells were incubated with 200 µl of warmed trypsin-EDTA (0.05%) for 1 minute at 37°C (or until the cells had visibly lifted). After 1 minute, the bottom of the flask was gently tapped to see if the cells had lifted. The reaction was quenched with 4 mL of Stage II Medium (prepared with 15% hiFBS). The contents of 6 wells were combined in a 50 mL falcon tube and gently triturated 5 – 6 times to form a single cell suspension. The cells were re-plated onto fresh Matrigel-coated plates. A complete media change was performed the following morning with fresh Stage II medium.
**Accutase**	Old culture media was aspirated and discarded from each well. Cells were incubated with 200 µl of Accutase for 5 minutes at 37°C (or until the cells had visibly lifted). After 1 minute, the bottom of the flask was gently tapped to see if the cells had lifted. The reaction was quenched with 4 mL of Stage II Medium. The contents of 6 wells were combined in a 50 mL falcon tube and gently triturated 5 – 6 times to form a single cell suspension. The cells were re-plated onto fresh Matrigel-coated plates. A complete media change was performed the following morning with fresh Stage II medium.
**Dispase**	Dispase was pre-warmed to room temperature prior to use. Old culture media was aspirated and discarded from each well. Cells were incubated with 200 µl of Dispase for 3 minutes at 37°C (or until the cells had visibly lifted). After 1 minute, the bottom of the flask was gently tapped to see if the cells had lifted. The reaction was quenched with 4 mL of Stage II Medium. The contents of 6 wells were combined in a 50 mL falcon tube and gently triturated 5 – 6 times to form a single cell suspension. The cells were re-plated onto fresh Matrigel-coated plates. A complete media change was performed the following morning with fresh Stage II medium.
**Versene**	Old culture media was aspirated and discarded from each well. Cells were incubated with 200 µl of Versene for 5 minutes at 37°C (or until the cells had visibly lifted). After 1 minute, the bottom of the flask was gently tapped to see if the cells had lifted. The reaction was quenched with 4 mL of Stage II Medium. The contents of 6 wells were combined in a 50 mL falcon tube and gently triturated 5 – 6 times to form a single cell suspension. The cells were re-plated onto fresh Matrigel-coated plates. A complete media change was performed the following morning with fresh Stage II medium.
**GCDR**	Old culture media was aspirated and discarded from each well. Cells were incubated with 200 µl of GCDR for 5 minutes at room temperature (or until the cells had visibly lifted). After 1 minute, the bottom of the flask was gently tapped to see if the cells had lifted. The reaction was quenched with 4 mL of Stage II Medium. The contents of 6 wells were combined in a 50 mL falcon tube and gently triturated 5 – 6 times to form a single cell suspension. The cells were re-plated onto fresh Matrigel-coated plates. A complete media change was performed the following morning with fresh Stage II medium

Supplementary information – FIJI script for analysis of cell occupied area in five regions of the well

```
// Setup
setOption("ExpandableArrays", true);

// Generate a prompt for the user to select the directory that contains the images to be processed
dir = getDirectory("Choose Nuclei image directory");
// Create a list of all the files in that directory
list = getFileList(dir);

Table.create("OverallResults");

// Loop over every file in the list of files
for (i=0; i<list.length; i++) {
		// We only want to open image files. This IF statement checks the file extension to see if the file is a ".png"
		// Can be altered for other image extensions
		// If the file is a ".png", then the image is processed, otherwise the file is skipped
		if (endsWith(list[i], ".png"))
				processFile(dir, list[i]);
		else {
				continue; //Ignore sub directories, and all files that are not ".png" files
		}
}

// Output the results in a '.csv' file in the same directory as the images
Table.update;
saveAs("Results", dir + "OverallResults.csv");
close("OverallResults.csv");

// This function does all the processing on an image. Input: 1) path to the image directory. 2) filename
function processFile(dir, filename) {

		// Close any images that are open, clear the Results Window, and clear the Log window/
		// This makes sure we start with a clean slate
		run("Close All");
		run("Clear Results");
		print("\\Clear");

		// Open the image
		open(dir + filename);

		// Save the name of the file, but without the .png extension. This is useful later on when saving files
		dotIndex = indexOf(filename, ".");
		basefilename = substring(filename, 0, dotIndex);

		// Create a table to store the results
		Table.set("Name", Table.size, basefilename, "OverallResults");

		// Rename the image window. It helps to standardise the names of the images windows to
		// keep track of all the images. Especially when processing in a loop.
		rename("original");

		// Convert the image to an 8-bit image. This is necessary for thresholding.
		run("8-bit");
		run("Gamma...", "value=0.50");

		// Create a copy of the image so we can do a background subtraction to correct for the uneven background
		run("Duplicate...", "title=bs"); // Give the image the name bs to help keep track
		// Perform background subtraction. The radius should be larger than the size in pixels of the nuclei in the image
		// Alter radius depending on resolution and magnification
		run("Subtract Background...", "rolling=150");

		// Variables that are going to be used as arrays need to be defined as arrays before they are used.
		// Note: These arrays are not a fixed size because of the setOption("ExpandableArrays", true); option at the top
		diameter = newArray;
		left = newArray;
		top = newArray;

		// Get the size of the image
		width = getWidth;
		height = getHeight;

		// We are going to define an ROI for the well based on the image size. widthOffset is a buffer in case the well
```

```
// does not react the border of the images.
// The well is assumed to be centered in the image.
// How many pixels on the top/bottom/left/right of the image should be excluded because they are not part of the well.
// User defined. Better to be slightly larger than needed.
widthOffset = 200;

// How many circles do you want to divide the well into?
nCircles = 2;

// Calculate the radius of the largest and smallest circles
// All the other radii are caluclated from minRadius
maxRadius = (width - (2 * widthOffset)) / 2;
minRadius = maxRadius / ((nCircles*2) - 1);

// Calculate the diameters of all the circles
// Circles are created from an Oval ROI. This needs the left and top positions of the oval, as well as the diameter.
// Assume the centre of the circle is in the middle of the image.
xCentre = width / 2;
yCentre = height / 2;
for (i = 0; i < nCircles; i++) {
		diameter[i] = (minRadius * ((i+1)*2 - 1)) * 2;
		left[i] = xCentre - (diameter[i] / 2);
		top[i] = yCentre - (diameter[i] / 2);
}

// Make masks for ring areas. Each mask will go into a new image
for (i = 0; i < nCircles; i++) {

		// Create a blank (black) image for the ring mask to go in
		// Name image based on 0...nCircles with 0 being the centre circle
		newImage("r"+i, "8-bit black", width, height, 1);

		// Create a circle ROI. Fill it with white
		makeOval(left[i], top[i], diameter[i], diameter[i]);
		setForegroundColor(255, 255, 255);
		run("Fill", "slice");

		// We are creating 'Ring' ROIs so we need to remove the inner part of the ring/
		// I.e. fill the previous ROI with black.
		// This is not necessary for the innermost ROI so skip with IF.
		if (i > 0) {
				makeOval(left[i-1], top[i-1], diameter[i-1], diameter[i-1]);
				setForegroundColor(0, 0, 0);
				run("Fill", "slice");
		}
		// Make sure we have deselected the ROIs we creates
		run("Select None");
}

// Make masks for quadrant areas
// Top Left
newImage("s0", "8-bit black", width, height, 1);
makeRectangle(0, 0, width/2, height/2);
setForegroundColor(255, 255, 255);
run("Fill", "slice");
run("Select None");

// Top Right
newImage("s1", "8-bit black", width, height, 1);
makeRectangle(width/2, 0, width/2, height/2);
setForegroundColor(255, 255, 255);
run("Fill", "slice");
run("Select None");

// Bottom Left
newImage("s2", "8-bit black", width, height, 1);
makeRectangle(0, height/2, width/2, height/2);
setForegroundColor(255, 255, 255);
run("Fill", "slice");
run("Select None");

// Bottom Right
newImage("s3", "8-bit black", width, height, 1);
makeRectangle(width/2, height/2, width/2, height/2);
setForegroundColor(255, 255, 255);
```

```
	run("Fill", "slice");
	run("Select None");

	// Combing quadrant and ring masks to make ring quadrants
	selectWindow("r0");
	rename("0");
	for (i = 0; i < 4; i++) {
		imageCalculator("AND create", "r1", "s"+i);
		rename(""+(i+1));
	}

	// Create a copy of the background subtracted image to create a threshold image
	selectWindow("bs");
	// Remove everything outside well. Not strictly necessary as we only analyse within ROIs
	makeOval(left[left.length-1], top[top.length-1], diameter[diameter.length-1], diameter[diameter.length-1]);
	run("Make Inverse");
	setForegroundColor(0, 0, 0);
	run("Fill", "slice");
	run("Select None");

	// Threshold based on values in well only
	run("Duplicate...", "title=threshold");
	makeOval(left[left.length-1], top[top.length-1], diameter[diameter.length-1], diameter[diameter.length-1]);
	setAutoThreshold("Otsu dark no-reset"); // Set threshold type
	setOption("BlackBackground", true); // Important setting
	run("Convert to Mask"); // Calculate the threshold

	// Create image with ROIs overlaid
	selectWindow("threshold");
	run("Analyze Particles...", "clear include add");
	selectWindow("bs");
	roiManager("Show All without labels");
	run("Flatten");
	rename("bs_roi");

	// Measure area of ring quadrants and centre
	circleArea = newArray(); // Define array to sture data
	for (i = 0; i < 5; i++) {
		selectWindow(i); // Select the image window that contains the first ring ROI
		run("Select All"); // Select the entire image
		// Measure the sum of all the pixel values.
		// The image is binary so we know that the image only contains black (0) and white pixels (255).
		// If we calculate the sum of the brightness values of all the pixels in the image, then
		// divide by 255, that tells us how many pixels are white, i.e. the area of white pixels
		run("Set Measurements...", "integrated redirect=None decimal=3");
		run("Measure");
		run("Select None");
		circleArea[i] = getResult("IntDen", i) / 255;
	}
	run("Clear Results"); // remove the results from the results window. Necessary to use the getResult function with the 0, 1... row
indicies

	// Measure area of cells. Same concept as above.
	cellArea = newArray();
	percentArea = newArray();
	for (i = 0; i < 5; i++) {
		// Apply mask
		imageCalculator("AND create", "threshold", ""+i);
		selectWindow("Result of threshold");
		rename("thresh"+i);

		run("Select All");
		run("Set Measurements...", "integrated redirect=None decimal=3");
		run("Measure");
		run("Select None");
		cellArea[i] = getResult("IntDen", i) / 255;
		percentArea[i] = cellArea[i] / circleArea[i] * 100;

		Table.set(""+i, Table.size-1, percentArea[i], "OverallResults");
	}

	// Print Results to the Log Window
	print("ID, CircleArea, CellArea, %CellArea");
	for (i = 0; i < 5; i++) {
		print(i + ", " + circleArea[i] + ", " + cellArea[i] + ", " + percentArea[i]);
```

```
	}

	// Create a folder to save all the images we created
	output_folder = dir + basefilename + "\\";
	File.makeDirectory(output_folder);

	// Save all the images. Useful for debugging
	// Save the masks and the masked cell areas
	for (i = 0; i < 5; i++) {
		selectWindow(i);
		saveAs("png",  output_folder + basefilename + "_Area" + i + ".png");

		selectWindow("thresh"+i);
		saveAs("png",  output_folder + basefilename + "_NucleiThresh" + i + ".png");
	}
	selectWindow("bs"); // Save the background subtracted image
	saveAs("png",  output_folder + basefilename + "_bs" + ".png");
	selectWindow("bs_roi"); // Save the background subtracted image with nuclei outlines
	saveAs("png",  output_folder + basefilename + "_bsroi" + ".png");
	selectWindow("threshold"); // Save the thresholded image
	saveAs("png",  output_folder + basefilename + "_NucleiThresh" + ".png");

	// Save the Log Window to a text tile
	selectWindow("Log");
	saveAs("Text", output_folder + basefilename + ".txt");

	// Close any images that are open, clear the Results Window, and clear the Log window
	run("Close All");
	run("Clear Results");
	print("\\Clear");
	selectWindow("Results");
	run("Close");

}
```
